# Arsenic targets Pin1 and cooperates with retinoic acid to inhibit cancer-driving pathways and tumor-initiating cells

**DOI:** 10.1038/s41467-018-05402-2

**Published:** 2018-08-09

**Authors:** Shingo Kozono, Yu-Min Lin, Hyuk-Soo Seo, Benika Pinch, Xiaolan Lian, Chenxi Qiu, Megan K. Herbert, Chun-Hau Chen, Li Tan, Ziang Jeff Gao, Walter Massefski, Zainab M. Doctor, Brian P. Jackson, Yuanzhong Chen, Sirano Dhe-Paganon, Kun Ping Lu, Xiao Zhen Zhou

**Affiliations:** 1 Department of Medicine, Division of Translational Therapeutics, Beth Israel Deaconess Medical Center, Harvard Medical School, Boston, MA 02215 USA; 2 Cancer Research Institute, Beth Israel Deaconess Medical Center, Harvard Medical School, Boston, MA 02215 USA; 3 Department of Cancer Biology, Dana Farber Cancer Institute, Boston, MA 02115 USA; 4 Department of Chemistry and Chemical Biology, Harvard University, Cambridge, MA 02138 USA; 5 Fujian Institute of Hematology, Fujian Provincial Key Laboratory on Hematology, Fujian Medical University Union Hospital, Fuzhou, Fujian 350108 China; 6 Fujian Key Laboratory for Translational Research in Cancer and Neurodegenerative Diseases, Institute for Translational Medicine, Fujian Medical University, Fuzhou, Fujian 350108 China; 7 Trace Element Analysis Lab, Dartmouth College, Hanover, NH 03755 USA; 8 Broad Institute of MIT and Harvard, Cambridge, MA 02142 USA

## Abstract

Arsenic trioxide (ATO) and all-*trans* retinoic acid (ATRA) combination safely cures fatal acute promyelocytic leukemia, but their mechanisms of action and efficacy are not fully understood. ATRA inhibits leukemia, breast, and liver cancer by targeting isomerase Pin1, a master regulator of oncogenic signaling networks. Here we show that ATO targets Pin1 and cooperates with ATRA to exert potent anticancer activity. ATO inhibits and degrades Pin1, and suppresses its oncogenic function by noncovalent binding to Pin1’s active site. ATRA increases cellular ATO uptake through upregulating aquaporin-9. ATO and ATRA, at clinically safe doses, cooperatively ablate Pin1 to block numerous cancer-driving pathways and inhibit the growth of triple-negative breast cancer cells and tumor-initiating cells in cell and animal models including patient-derived orthotopic xenografts, like Pin1 knockout, which is substantiated by comprehensive protein and microRNA analyses. Thus, synergistic targeting of Pin1 by ATO and ATRA offers an attractive approach to combating breast and other cancers.

## Abstract (web summary)

Arsenic trioxide and all-*trans* retinoic acid combination safely cures human acute promyelocytic leukemia. Here, the authors show that this combination has potent anticancer activity in triple negative breast cancer by cooperatively targeting Pin1, a master  regulator of oncogenic signaling networks, to eliminate cancer stem cells.

## Introduction

Aggressive solid tumors are often resistant to targeted therapies aiming at blocking individual pathways largely due to the simultaneous activation of a wide range of interactive and/or redundant pathways and/or oncogene switching^1,2^. To meet this challenge, it has been proposed to use various “-omic” techniques to identify all activated pathways in each tumor and then to use a cocktail of drugs to inhibit individual targets/pathways identified^1,2^. However, individual cancer cells within a tumor are highly heterogeneous and evolving^3^, and many cancer drivers, notably transcription factors, are non-druggable^1,2^. Moreover, current therapies do not effectively target tumor-initiating cells/cancer stem cells (TICs/CSCs), which are suggested to be responsible for tumor initiation, growth, metastasis, and drug resistance^4,5^. Identifying and inhibiting single targets driving multiple signaling mechanisms in cancer cells and TICs may offer a promising strategy to overcome drug resistance^6,7^.

As one of the oldest drugs, arsenic has been used to treat a variety of ailments, ranging from infection to cancer^8,9^. In the nineteenth century, arsenic, in the form of Fowler’s solution, served as an anti-leukemic remedy until its replacement by radiation and chemotherapy in the early twentieth century^8,9^. In 1970s, the use of arsenic to treat cancer resurfaced with the discovery of the arsenic-rich traditional Chinese medicine called “Ai-Ling #1” (magic bullet for cancers #1) for treating acute promyelocytic leukemia (APL) and other cancers^8,9^. Arsenic trioxide (ATO) was identified as the active component of Ai-Ling #1 and it was approved by Food and Drug Administration (FDA) for APL treatment in 1995^8,9^. ATO, when combined with all-*trans* retinoic acid (ATRA), a vitamin A derivative, has transformed APL from being highly fatal to highly curable, with minimal toxicity even in children^10–12^. The drug mechanism has long been attributed to their combined ability to induce degradation of the disease-causing oncoprotein promyelocytic leukemia-retinoic acid receptor α (PML-RARα) by acting on the two fusion partners; ATO covalently interacts with Cys in PML, whereas ATRA activates RARα receptor to induce cell differentiation^10–12^. However, their mechanisms of action and efficacy, especially in other cancers, remain elusive.

ATO has also shown efficacy against other hematologic malignancies and various solid tumors including breast and liver cancer^9,13^. Epidemiological studies have shown that although drinking water contamination with low ATO levels might increase cancer risk^14^, high level ATO drinking water contamination markedly reduces overall breast cancer mortality in the large affected population by over 50% during a 15-year contaminating period and in women under 60 by 70%^15^. However, the mechanisms mediating these anticancer effects of ATO are not clear. This question is important because ATO, at therapeutic doses, has an excellent safety profile for treating APL even in children^10–12^, although it has notorious toxicity at high doses due to its covalent binding to cellular targets^9,16^.

Similarly, regular ATRA, even with a half-life of 45 min, has moderate but detectable efficacy against solid tumors in clinical trials, but its second and third generation supposedly much more potent analogs to target RARs or RXRs show little efficacy in clinical trials^17–19^. Even in APL, ATRA’s ability to activate RARs and induce leukemia cell differentiation can be uncoupled from its activity to induce PML-RARα degradation, inhibit APL stem cells, and treat APL^20,21^. Moreover, ATRA’s ability to activate RARs cannot explain its activity to destabilize oncoproteins^22^ and stabilize tumor suppressors^23^. These puzzling findings may be explained by our recent unexpected discovery of ATRA, but its second-generation and third-generation analogs, as an inhibitor of Pin1^24^, a major common regulator of oncogenic signaling networks^7,25^.

A central signaling mechanism in regulating numerous oncoproteins and tumor suppressors is Pro-directed Ser/Thr phosphorylation (pSer/Thr-Pro) that is regulated by many kinases and phosphatases^7,26^, and further controlled by a single proline isomerase Pin1^6,7,25^. Numerous lines of evidence suggest that Pin1 is a critical “driver” and a unique drug target in cancer^6,7,25^. Pin1 is hyperactivated in most human cancers and correlates with poor clinical outcome^6,7,25^, whereas humans with genetic polymorphisms that reduce PIN1 expression have a lower risk for multiple cancers^6,7,25^. Pin1 knockout (−/−, KO) mice are highly resistant to tumorigenesis even amid overexpression of oncogenes such as HER2^27^, RAS^27^, Myc^28^, or after mutation^29^ or ablation^30^ of tumor suppressors such as p53. Conversely, Pin1 overexpression disrupts cell cycle coordination leading to chromosome instability and cancer development^31^. Pin1 activates at least 43 oncoproteins, inactivates over 20 tumor suppressors, and downregulates global microRNAs, acting as the “master” post-phosphorylation regulator of oncogenic signaling networks^7,25,32^. Moreover, Pin1 is highly enriched in breast TICs/CSCs to drive their self-renewal and tumor initiation^33–35^. Finally, Pin1 has a critical role in viral, bacterial, and parasitic infections and their related malignancies^36^. Notably, Pin1−/− mice display no obvious defects over half lifespan^7,37^. Thus, targeting Pin1 represents a novel non-toxic strategy to simultaneously block multiple cancer-driving pathways and also eliminate TICs^7,25^. However, Pin1 inhibitors identified previously lacked the specificity, potency, and/or cell permeability^38^.

Our recent mechanism-based drug screens have identified ATRA as a Pin1 inhibitor^24^. ATRA binds, inhibits, and induces Pin1 degradation, thereby destabilizing its substrate PML-RARα and treating APL in cell and animal models and human patients^24^. ATRA-induced Pin1 ablation also exerts antitumor activity against breast cancer by blocking multiple oncogenic pathways. The ability of ATRA to inhibit Pin1 function has been confirmed in breast cancer^24,39^ and liver cancer^24,40,41^ even using a different ATRA controlled release formulation^42^, and acute myeloid leukemia (AML)^43^, as well as in lupus^44^ and asthma^45^. However, regular ATRA formulation has a half-life of only 45 min in humans^46^ and biodegrabable longer half-life of ATRA formulations that might be used in humans are under development^42^. Thus, more effective and clinically usable Pin1 inhibitors are urgently needed.

In this manuscript, we report the surprising findings that ATO inhibits and induces Pin1 degradation and suppresses cancer cell growth via noncovalently binding to the Pin1 active site, as corraborated by nuclear magnetic resonance (NMR) and co-crystal structure. ATO, at clinically relevant and safe doses, ablates Pin1 to inactivate multiple oncoproteins and activate many tumor suppressors and global microRNAs, as well as inhibit triple-negative breast cancer (TNBC) tumor growth. Disrupting the ability of Pin1 to bind to ATO results in ATO resistance in vitro and in vivo. Moreover, the anticancer effects of ATO are potently amplified by co-treatment with ATRA, which induces aquaporin-9 (AQP9) to increase cellular ATO uptake, in addition to directly inhibiting and degrading Pin1. Consequently, ATO and ATRA work cooperatively to ablate Pin1, thereby blocking multiple oncogenic pathways and eliminating TICs and their drug resistance in TNBC in human cells and in animal models including patient-derived orthotopic xenografts (PDOXs). This ATO-ATRA cooperative phenotype closely resembles Pin1 CRISPR KO, which is also substantiated by comprehensive analyses of protein and microRNA expression. Thus, Pin1 is a novel drug target for ATO, and synergistic targeting of Pin1 by ATO and ATRA offers an attractive approach to block multiple cancer-driving pathways and eliminate TICs, which are the two major sources of drug resistance in current cancer therapy.

## Results

### ATO induces Pin1 degradation and inhibits cell growth

**Fig. 1:**
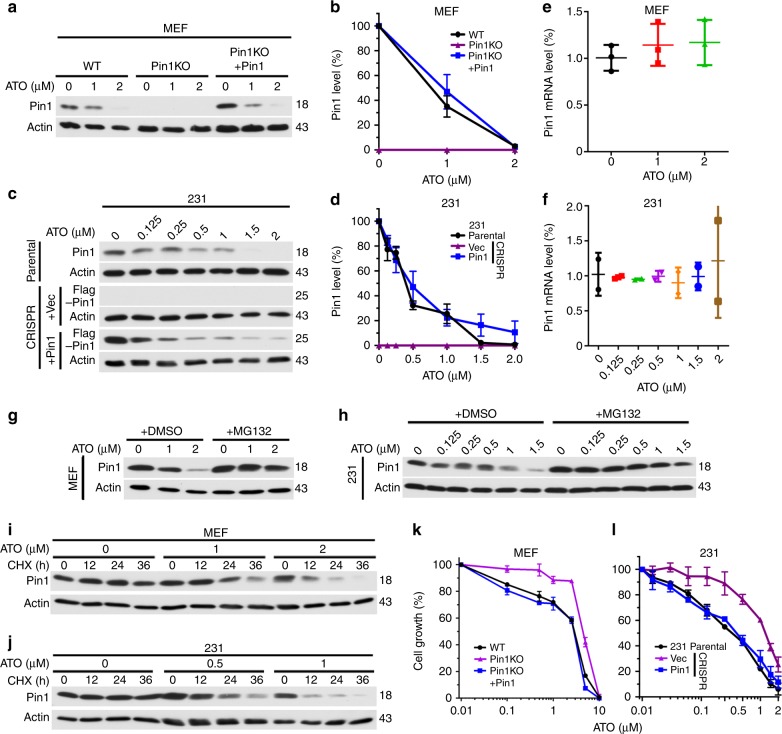
ATO induces Pin1 degradation and inhibits cell growth at clinically relevant concentrations. **a**–**d** ATO dose-dependently reduces Pin1 in MEFs (**a**, **b**) and 231 cells (**c**, **d**). Pin1 WT and KO MEFs or 231 cells, or Pin1 KO MEFs or 231 cells reconstituted with Pin1 were treated with different concentrations of ATO for 3 days, followed by Pin1 immunoblot (**a**, **d**) and quantification (**b**, **d**). **e**, **f** ATO does not affect Pin1 mRNA levels in MEFs (**e**) and 231 cells (**f**). MEFs and 231 cells were treated with different concentrations of ATO for 3 days, followed by assaying Pin1 mRNA using real-time PCR analysis. **g**, **h** ATO-induced Pin1 downregulation is rescued by proteasome inhibition in MEFs (**g**) and 231 cells (**h**). MEFs or 231 cells were treated with different concentrations of ATO in the absence or presence of MG132, followed by Pin1 immunoblot. **i**, **j** ATO dose-dependently reduces Pin1 protein stability in MEFs (**i**) and 231 cells (**j**). MEFs or 231 cells were treated with different concentrations of ATO in the absence or presence of cycloheximide, followed by Pin1 immunoblot. **k**, **l** ATO dose-dependently inhibits cell growth of Pin1 WT, but not Pin1 KO MEFs and 231 cells, which can be rescued by re-expressing Pin1. Pin1 WT and KO MEFs (**k**) or 231 cells (**l**) or Pin1 KO MEFs or 231 cells reconstituted with Pin1 were treated with different concentrations of ATO for 3 days, followed by assaying cell growth. The results are expressed as mean ± S.D. and the *P* values (*P < 0.05, **P < 0.01, ***P < 0.001) were determined by ANOVA test

ATO, especially in combination with ATRA, effectively cures the fatal disease APL^10–12^. Since ATRA inhibits APL, AML, breast cancer, and liver cancer by targeting Pin1^24,40–43^, we wondered whether ATO has any effects on Pin1. Using concentrations (0.1–2 µM) that have widely and safely been used in APL cells^47–49^, we surprisingly found that ATO dose-dependently downregulated Pin1 protein levels in mouse embryonic fibroblasts (MEFs) (Fig. 1a, b), human TNBC MDA-MB-231 (231) cells (Fig. 1c, d) and many other breast cancer cells (see below). ATO had no effects on Pin1 mRNA levels (Fig. 1e, f), and ATO-induced Pin1 degradation was rescued by the proteasome inhibitor MG132 (Fig. 1g, h and Supplementary Fig. 1a, b). Moreover, ATO dose-dependently reduced Pin1’s protein half-life in MEFs and 231 cells (Fig. 1i, j and Supplementary Fig. 1c, d). Thus, ATO induces proteasome-dependent Pin1 degradation.

To determine whether ATO inhibits Pin1 function in cells, we examined its effects on the growth of Pin1 KO (*Pin1*−/−) and wild-type (WT, *Pin1*+/+) MEFs, which display a differential response to Pin1 inhibition by ATRA^24^. ATO dose-dependently inhibited Pin1 WT MEF growth, but was less effective against Pin1 KO MEF growth, and the effect was restored by stably re-expressing Pin1 (Fig. 1k). To confirm these results, we generated Pin1 KO 231 cells using the CRISPR-Cas9 system, and verified them using DNA sequencing and protein analysis (Fig. 1c). Again, Pin1 CRISPR KO cells were more resistant to ATO, except when Pin1 levels were brought back to endogenous levels using a lentiviral vector containing an altered Kozak sequence (Fig. 1c, l)^34^. Thus, ATO inhibition of Pin1 contributes to its anti-proliferative effects.

### ATO directly and noncovalently binds to and inhibits Pin1

**Fig. 2:**
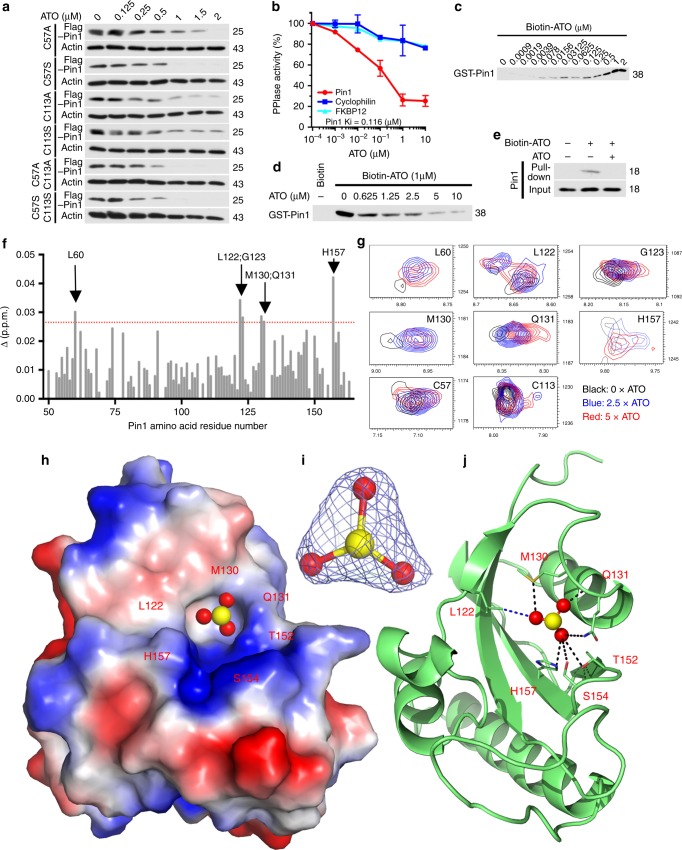
ATO directly binds to specific residues in the Pin1 active site via a previously unknown mechanism. **a** Cys residues in Pin1 are not required for ATO to degrade Pin1. Pin1 KO cells stably expressing Cys single or double Pin1 Ala or Ser mutants were treated with different concentrations of ATO, followed by Pin1 immunoblot. **b** ATO inhibits PPIase activity of Pin1, but not Cyp18 or FKBP12. Pin1, Cyp18, or FKBP12 was incubated with different concentrations of ATO, followed by chymotrypsin-coupled PPIase assay. **c** Biotin-ATO binds to Pin1. Pin1 was incubated with biotin-ATO, followed by isolating biotin-ATO-bound Pin1 for immunoblot (top) and plotting against ATO concentrations (bottom). **d** ATO binding to recombinant Pin1 can be competed by ATO. Biotin-ATO was incubated with recombinant Pin1, followed by incubation with ATO before subjecting biotin-ATO pulldown assay and Pin1 immunoblot. **e** ATO binding to cellular Pin1 can be competed by ATO. The 231 cells were treated with or without ATO and then subjected to biotin-ATO pulldown assay, followed by Pin1 immunoblot, along with Pin1 imputs. **f**, **g** NMR analysis of ATO-Pin1 binding. Weighted average chemical shift difference Δ of 15N-Pin1 upon addition of 5× ATO was calculated as |Δ*H*| + (1/5)|Δ*N*| in p.p.m. and plotted as a function of residue number (**f**). Upon addition of 2.5× or 5× ATO to Pin1, total six residues in Pin1 show significant chemical shift changes at both ATO concentrations, with two Cys that show no obvious chemical shift changes being highlighted (**g**). **h**–**j** The co-crystal structure of the Pin1-ATO complex. ATO was mixed and co-crystallized with 500 µM Pin1, followed by collecting diffraction data at synchrotron beamline 24ID using and integrating and scaling data sets using XDS. Identical novel trigonal electron density in shape was noted at the Pin1 active site in multiple co-crystals (**h**, **i**). The apexes of this electron density are positioned within hydrogen bonding distances (dark green) of side chains from Met130, Gln131, Thr152, Ser154, and His157 and within van der Waals distance (blue) of side chain from Leu122 in the Pin1 active site (**j**)

**Fig. 3:**
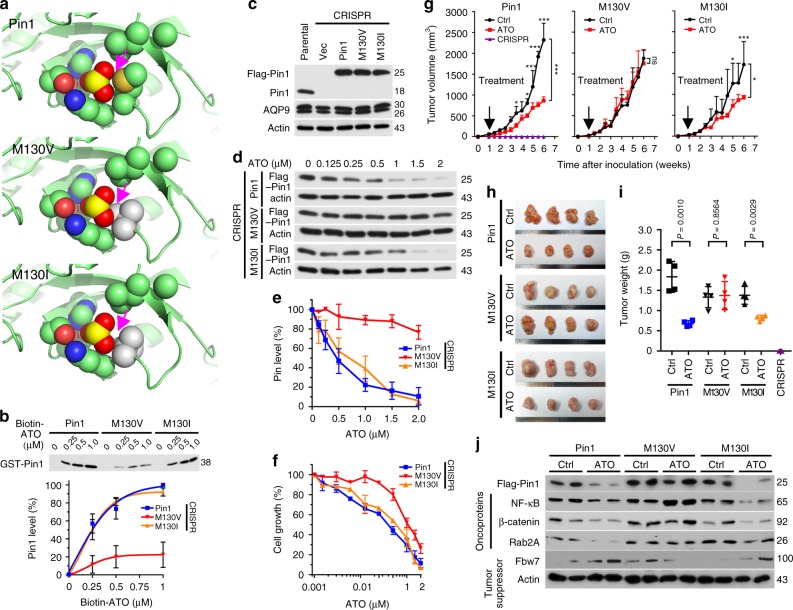
Disrupting ATO binding to Pin1 impairs its ability to induce Pin1 degradation and to inhibit breast cancer tumor growth. **a** The co-crystal structure of ATO and Pin1 complexes suggests that the M130V, but not M130I Pin1 mutation, would prevent ATO from binding to the Pin1 active site (arrows). **b** Biotin-ATO binds to the M130I, but not M130V Pin1 mutant. Pin1 and its mutants were incubated with different concentrations of biotinylated ATO, followed by isolating biotin-ATO-bound Pin1 or its mutants using NeutrAvidin beads. ATO-bound Pin1 were detected by immunoblot and plotted against ATO concentrations. **c**–**f** The M130V, but not M130I, Pin1 mutation impairs ATO’s ability to induce Pin1 degradation and inhibit cell growth. Pin1 CRISPR cells stably expressing Pin1 or its M130V or M130I mutant (**c**) were treated with ATO, followed by assaying Pin1 levels (**d**, **e**) or cell growth (**f**). **g**–**j** The M130V, but not M130I Pin1, mutation impairs the ability of ATO to inhibit tumor growth in mice. Female nude mice were flank inoculated with 1 × 10^6^ Pin1 CRISPR cells that stably re-expressed Pin1, or its M130V or M130I mutant, and 1 week later, treated with ATO (2 mg/kg, i.p., 3 times/week). Tumor sizes were weekly measured (**g**) and mice were sacrificed after 5 weeks to collect tumor tissues (**h**) and measure their weights (**i**), as well as their expression of Pin1 and selected Pin1 substrate (**j**). The results are expressed as mean ± S.D. and the *P* values (*P < 0.05, **P < 0.01, ***P < 0.001) were determined by ANOVA test. *n* = 4–6 mice

It has been shown that ATO exerts its cellular effects by covalently interacting with vicinal Cys residues in its targets including PML-RARα^9,16,50,51^. Pin1 has two Cys residues, Cys113 and Cys57. To examine whether they are required for ATO to induce Pin1 degradation, we mutated them to Ala or Ser individually or in combination, and stably expressed the Pin1 mutants in Pin1 CRISPR KO 231 cells at levels similar to endogenous Pin1 (Fig. 2a). ATO equally degraded the single and double Pin1 Cys mutants (Fig. 2a) as WT protein (Figs. 1c, 3c), indicating that Pin1’s Cys residues are not necessary for ATO-induced Pin1 degradation.

To examine whether ATO would affect Pin1 catalytic activity, we used the standard chymotrypsin-coupled peptidyl-prolyl isomerase (PPIase) assay^52^. ATO dose-dependently inhibited Pin1 PPIase activity (Ki = 0.116 µM) (Fig. 2b), which is phosphorylation-specific, but had minimal effects on cyclophilin (Cyp18) or FKBP12 (Fig. 2b), members of the two major non-phosphorylation-specific PPIase families, cyclophilins and FK506-binding proteins^52^. To examine whether ATO would directly bind to Pin1 and to determine its binding affinity, we synthesized a biotinylated arsenate compound (biotin-ATO) and performed a binding assay using recombinant Pin1. Biotin-ATO directly bound to Pin1 in a concentration-dependent manner (apparent Kd = 0.238 µM) (Fig. 2c), consistent with the PPIase results (Fig. 2b), and was dose-dependently competed by ATO (Fig. 2d). Biotin-ATO also pulled down Pin1 from 231 cells, and binding was competed by ATO (Fig. 2e). Thus, ATO directly binds and inhibits Pin1 catalytic activity with an affinity of 0.1–0.2 µM.

To understand how ATO binds and inhibits Pin1 catalytic activity, we assessed the dynamics of ATO binding to ^15^N-labeled Pin1 PPIase domain using nuclear magnetic resonance (NMR) spectroscopy. Upon addition of ATO, select cross-peaks in the ^1^H-^15^N HSQC spectrum of Pin1 shifted and broadened in a dose-dependent manner, indicating binding. The residues perturbed upon ATO binding were located in the Pin1 active site, with particularly significant changes observed for Leu60, Leu122, Gly123, Met130, Gln131, and His157 (Fig. 2f, g and Supplementary Fig. 2a). Notably, ATO titration did not affect the cross-peaks for Cys57 or Cys113 (Fig. 2f, g and Supplementary Fig. 2a), further supporting that Pin1 binding of ATO is not Cys-mediated.

A search in the NCBI structure database showed several dozens of arsenic–protein complexes with covalent interactions between arsenic compounds and vicinal Cys or Cys-like cofactors or functional groups in targets, as per the commonly known mechanism^16^. A similar covalent interaction has been proposed to mediate ATO binding to PML-RARα^50,51^. To explore our unexpected noncovalent binding mode of ATO to Pin1, we co-crystallized excess ATO with the Pin1 PPIase domain and refined the structure to 1.6 Å resolution with excellent statistics (Supplementary Table 1). We noted well-defined novel electron density in the prolyl binding pocket of the Pin1 active site that was trigonal in shape with significant Fo-Fc values at 4*σ* (Fig. 2h, i). Although anomalous signal at 1.0438 Å was weak, isomorphous Fo-Fo maps calculated from ATO-soaked and Apo data sets showed clear density for what appeared to be ATO with central arsenic density peak >6*σ*. The electron density was nicely situated within the Pin1 catalytic active site positioned within van der Waals or hydrogen bonding distances of Leu122, Met130, Gln131, Thr152, Ser154, and His157 (Fig. 2j and Supplementary Fig. 2b). This model of ATO binding was consistent with the degree of change in chemical shift for all backbone amides in Pin1 revealed by NMR analysis. Again, neither Cys57 nor C113 were close to the ATO-binding pocket. Thus, ATO inhibits and induces Pin1 degradation via a novel noncovalent mechanism, distinct from the previous action modes of ATO on PML-RARα and others^16,50,51^.

### Disruption of Pin1 binding to ATO leads to ATO resistance

To demonstrate the significance of the novel interaction between Pin1 and ATO, we sought to identify a Pin1 point mutant that would disrupt ATO binding and determine the importance of direct ATO-Pin1 binding in vitro and in vivo. Since most of the ATO-binding residues are also involved in binding of Pin1 to proline residue in its substrate^53^, we were careful to select a mutation that would not severely impair Pin1 enzymatic activity. Indeed, point substitutions at T152 or H157 almost completely inactivated Pin1 PPIase activity (Supplementary Fig. 2c, d). We did manage to generate a pair of enzymatically active Pin1 M130 mutants, albeit with slightly lower activity than the WT protein (Supplementary Fig. 2c, d), likely caused by altered proline binding of the substrate. The Pin1-ATO co-crystal structure predicted that an M130V mutation would disrupt ATO binding, whereas an M130I mutant would bind to ATO like the WT protein (Fig. 3a). Indeed, Pin1 M130I mutant-bound biotin-ATO with a similar affinity to the WT protein, whereas Pin1 M130V mutant had a much reduced affinity for Biotin-ATO (Fig. 3b). ATO dose-dependently inhibited the PPIase activity of Pin1 M130I, but not Pin1 M130V mutant (Supplementary Fig. 2e). Thus, the M130V, but not M130I, mutation in Pin1 disrupts Pin1 binding to ATO, as predicted.

If direct binding to Pin1 is critical for ATO to target Pin1 in TNBC, we would expect expression of the Pin1 M130V mutant in Pin1 KO cells to reduce the sensitivity to ATO in vitro and in vivo. To test this possibility, we stably expressed Pin1, Pin1 M130V, and M130I mutants in Pin1 CRISPR KO 231 cells at endogenous levels (Fig. 3c), and then assayed their response to ATO. Pin1 CRISPR KO cells were used to avoid the potential effects of endogenous Pin1. As expected, cells expressing the Pin1 M130V mutant showed impaired ATO-induced Pin1 degradation and inhibition of cell growth, whereas cells expressing the Pin1 M130I mutant behaved similarly to the WT protein (Fig. 3d–f). To confirm these results, we orthotopically xenografted Pin1 CRISPR KO 231 cells expressing Pin1 or its mutants into mice, and 1 week later when tumor growth was notable, the xenografted mice was treated with ATO at 2 mg/kg 3 times/week, a standard concentration that has widely and safely been used for treating APL in mouse models and human patients^47–49^. Pin1 CRISPR KO 231 cells failed to grow any tumors in mice (Fig. 3g–i), consistent with the findings that Pin1 KO mice are highly resistant to cancer development^27–30^. In contrast, tumors did develop in Pin1 CRISPR KO 231 cells expressing Pin1 or its M130I or M130V mutant, although the tumors of the Pin1 mutants were slightly smaller than WT Pin1 tumors (Fig. 3g–i), consistent with their lower PPIase activity (Supplementary Fig. 2c, d). Importantly, ATO treatment effectively inhibited the growth of tumors derived from Pin1 or its M130I mutant, but not at all from the M130V mutant (Fig. 3g–i). Moreover, ATO reduced the levels of Pin1 and its substrate oncoproteins such as NF-κB/p65^54^, β-catenin^55^, and Rab2A^34^, and increased the levels of Pin1 substrate tumor suppressors such as Fbw7^56^ in breast tumors derived from xenografts expressing Pin1 or M130I mutant, but not M130V mutant (Fig. 3j). Thus, ATO binding to Pin1 is essential for ATO to induce Pin1 degradation, block oncogenic pathways, and inhibit tumor growth.

### ATO uptake via AQP9 regulates its ability to inhibit Pin1

**Fig. 4:**
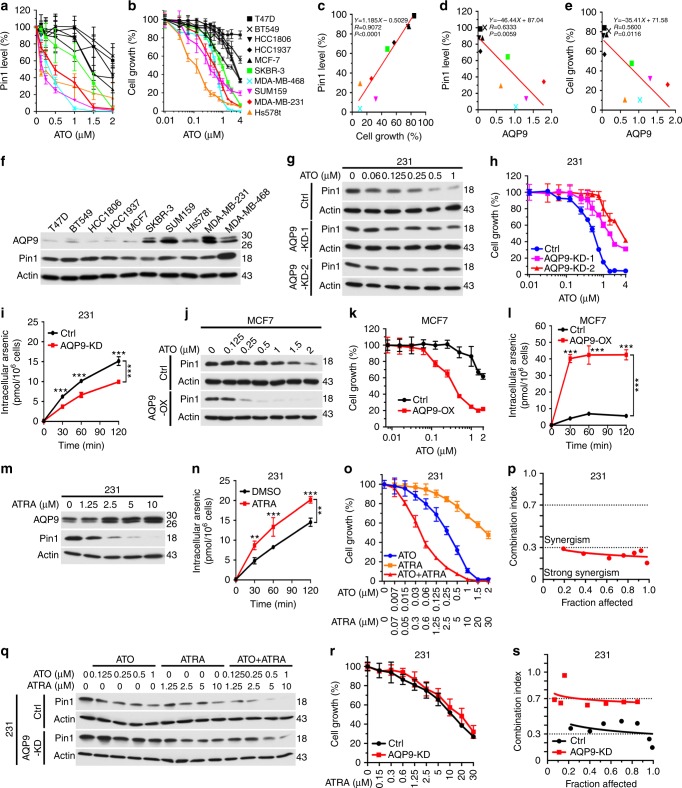
ATRA cooperates with ATO to induce Pin1 degradation and inhibit cancer cell growth by increasing cellular ATO uptake via the induction of AQP9 expression. **a**–**c** Correlation between the ability of ATO to induce Pin1 degradation and to inhibit cell growth. Ten human breast cancer cells were treated with ATO for 3 days, followed by Pin1 immunoblot (**a**) and counting cell numbers (**b**), and determining their correlation (**c**). **d**–**f** Correlation between AQP9 expression and the ability of ATO to degrade Pin1 and to inhibit cell growth. AQP9 expression were assayed by immunoblot (**f**) and their correlations with the ability of ATO to degrade Pin1 (**d**) and inhibit cell growth (**e**) were calculated using the data from **a**, **b**, and **f**. **g**–**i** AQP9 KD reduces ATO sensitivity in ATO-sensitive cells. Stable AQP9 KD 231 cells generated using two unrelated shRNA vectors were treated with different concentrations of ATO for 3 days, followed by assaying Pin1 levels (**g**) and cell growth (**h**), or with 1 µM ATO for different times, followed by assaying cellular ATO concentrations by ICP-Mass Spec (**i**). **j**–**l** AQP9 OX reverses ATO resistance in ATO-resistant cells. Stable AQP9 OX MCF7 cells were treated with different concentrations of ATO for 3 days, followed by assaying Pin1 levels (**j**), cell growth (**k**), or with 1 µM ATO for different times, followed by assaying cellular ATO concentrations by ICP-Mass Spec (**l**). **m**–**p** ATRA induces AQP9 protein expression, increases ATO uptake, and cooperates with ATO in inhibiting cell growth in TNBC cells. The 231 cells were treated with different concentrations of ATRA for 7 days, followed by AQP9 immunoblot (**m**) or with 1 µM ATO for different times before subjecting to ATO concentrations by ICP-Mass Spec (**n**), or with different concentrations of ATO and/or ATRA for 3 days, followed by counting cell number (**o**) and determining their synergy using CalcuSyn (**p**). **q**–**s** AQP9 KD abolishes ATRA cooperation with ATO, but does not affect ATRA sensitivity. Control or AQP9 KD 231 cells were treated with ATO and/or ATRA, followed by Pin1 immunoblot (**q**) and cell growth assay (**r**), followed by using CalcuSyn to calculate their synergy (**s**). The results are expressed as mean ± SD and the *P* values were determined by ANOVA test

To further support ATO’s potent anticancer activity via targeting Pin1, we examined the effects of ATO on cell growth using 10 different human breast cancer cell lines. Cells were treated with increasing concentrations of ATO and assessed for Pin1 levels (Fig. 4a and Supplementary Fig. 3a) and cell growth (Fig. 4b). ATO-induced Pin1 degradation was tightly and positively correlated with ATO-inhibited cell growth (Fig. 4c). However, ATO sensitivity was surprisingly variable among different cell lines. To identify the underlying mechanisms, we examined expression of AQP9, a membrane transporter that mediates cellular uptake of ATO known to correlate with ATO sensitivity in APL^57,58^. Indeed, AQP9 was readily detected in ATO-responsive cells, but not in ATO-resistant cells (Fig. 4f), with AQP9 expression being inversely correlated with Pin1 level and cell growth (Fig. 4d, e). Thus, ATO’s ability to inhibit breast cancer is positively correlated with Pin1 degradation and AQP9 expression.

To demonstrate the functional significance of AQP9 expression in determining ATO sensitivity, we stably knocked down AQP9 in two ATO-sensitive cells (Supplementary Fig. 3b) and overexpressed AQP9 in three ATO-resistant cells (Supplementary Fig. 3e). Two different AQP9 short hairpin RNA (shRNA) constructs effectively silenced AQP9 (Supplementary Fig. 3b), and also abrogated the ability of ATO to induce Pin1 degradation (Fig. 4g and Supplementary Fig. 3c) and inhibit cell growth (Fig. 4h and Supplementary Fig. 3d) in both cell lines, with shAQP9-2 being more effective. In contrast, AQP9 overexpression (Supplementary Fig. 3e) converted all three ATO-resistant cells to become ATO-sensitive cells in terms of Pin1 degradation (Fig. 4j and Supplementary Fig. 3f) and growth inhibition (Fig. 4k and Supplementary Fig. 3g). These results are further supported by measuring cellular ATO uptake using inductively coupled plasma mass spectrometry (ICP-MS). Whereas AQP9 knockdown (KD) reduced ATO uptake in ATO-sensitive cells (Fig. 4i), and AQP9 overexpression increased ATO uptake in ATO-resistant cells (Fig. 4l). Thus, ATO uptake via AQP9 regulates its ability to induce Pin1 degradation and inhibit cancer cells.

### ATO and ATRA cooperately inhibit Pin1 and oncogenic pathways

To demonstrate the cooperation and translational significance of ATO and ATRA in targeting Pin1 for treating cancers, we chose TNBC as a model system because unlike APL, which is basically cured by ATO and ATRA^10–12^, TNBC has the worst prognosis of all breast cancer subtypes and no targeted therapy is available^59^. Furthermore, Pin1 plays an essential oncogenic role in breast cancer^27,31,60,61^, and chemical ablation of Pin1 by ATRA exerts antitumor activity against TNBC^24^. Finally, as shown in APL^57,58^, ATRA dose-dependently increased both AQP9 mRNA (Supplementary Fig. 4a, b) and protein expression (Fig. 4m and Supplementary Fig. 4c) in TNBC cells, likely due to activation of the AQP9 promoter activity by ATRA, as shown by promoter reporter and mutagenesis analyses (Supplementary Fig. 4d). Moreover, ATRA and ATO combination increased time-dependent ATO uptake (Fig. 4n), and cooperately ablated Pin1 in two TNBC cells (Supplementary Fig. 5a, b).

To independently confirm the cooperative effects of ATO and ATRA on Pin1 levels, we established an in-cell enzyme-linked immunosorbent assay (ELISA) to quantify Pin1 protein levels after drug treatments, which correlated well with the Pin1 levels quantified using immunoblotting (Supplementary 5c–e). Importantly, the in-cell ELISA confirmed that, while either ATO or ATRA alone dose-dependently reduced Pin1 levels, their combination displayed strong synergy (Supplementary Fig. 5f), as calculated by the CalcuSyn program with the Chou–Talalay method^62^. As single agents, ATO and ATRA caused dose-dependent inhibition of cell growth in two TNBC cells, but their combination displayed synergistic effects (Fig. 4o, p and Supplementary Fig. 5g, h). To confirm the potential effects of ATRA on ATO response, we treated two AQP9 KD TNBC cells with either ATO, ATRA, or their combination. AQP9 KD did not affect the ability of ATRA to induce Pin1 degradation (Fig. 4q and Supplementary Fig. 5i) or inhibit cell growth (Fig. 4r and Supplementary Fig. 5j), but did largely abrogate its ability to synergize with ATO, which prevented additional Pin1 degradation (Fig. 4q and Supplementary Fig. 5i) and cell growth inhibition (Fig. 4s and Supplementary Fig. 5k). Thus, ATO cooperates with ATRA to promote Pin1 degradation and inhibit cell growth by inducing AQP9 expression in TNBC.

**Fig. 5:**
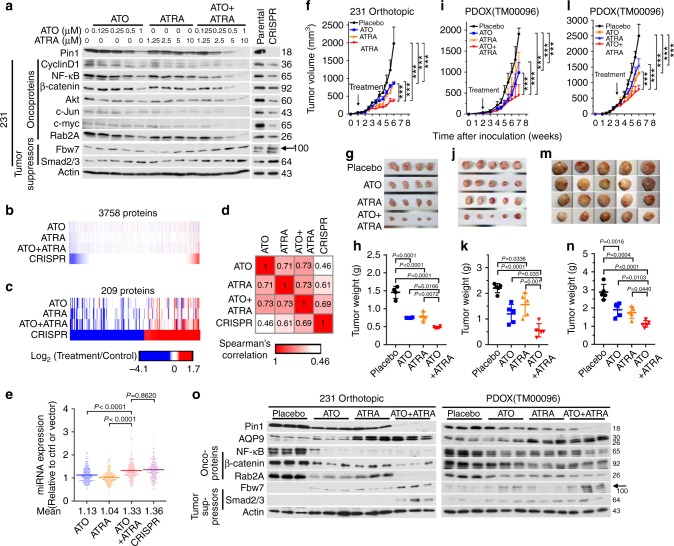
ATO and ATRA cooperatively ablate Pin1 and inhibit multiple Pin1-regulated oncogenic pathways and tumor growth in TNBC in vitro and in vivo including PDOXs. **a** ATO and ATRA cooperatively turn off many oncoproteins and on many tumor suppressors, like Pin1 KO. The 231 and 159 cells were treated with different concentrations of ATO and/or ATRA for 72 h, followed by IB, with Pin1 KO cells as controls. **b**–**d** ATO and ATRA cooperatively induced global protein expression like Pin1 KO. The 231 cells were treated with ATO and/or ATRA or DMSO for 72 h, followed by quantitative mass spectrometry analyses, with Pin1 KO 231 cells as a control. Three thousand seven hundred and fifty-eight proteins passed the abundance filter (**b**), and 209 proteins were altered by >1.5-fold (**c**). The log 2 transformed ratio of treated versus control was used to generate the heatmap in GENE-E. The Spearman's correlation matrix for the 209 altered proteins are shown and their *P* values are all below 2.2e−16, except *P* value for ATO and Pin1 KO being 3.5e−12 (**d**). **e** ATO and ATRA globally upregulates microRNA expression like Pin1 KO. MicroRNAs of ATO-treated and/or ATRA-treated 231 cells and Pin1 KO 231 cells were profiled by NanoString. Data are presented as relative to microRNA expression of DMSO-treated (Ctrl) 231 cells or vector CRISPR 231 cells through the dot density plot. The *P* values were determined by Student’s *t* test. **f**–**h** ATO and ATRA cooperatively inhibit tumor growth in TNBC 231 orthotopic xenografts. The 231 cells were transplanted into mammary fat pads, and 1 week later, treated with ATO and/or ATRA. Tumor sizes were measured (**f**) and mice were sacrificed after 6 weeks to collect tumor tissues (**g**) and measure their weights (**h**). **i**–**n** ATO and ATRA cooperatively inhibit tumor growth in TNBC PDOXs. TNBC patient-derived tumors were transplanted, followed by treating mice with ATO and/or ATRA 2–3 weeks after xenograft when tumors were notable (**i**–**k**) or reached about 360 mm^3^ (**l**–**n**). **o** ATRA induces AQP9 to cooperate with ATO to downregulate Pin1 and Pin1 oncogenic substrates and upregulate Pin1 tumor-suppressive substrates in human cells and PDOXs, assayed by immunoblot. The results are expressed as mean ± SD and the *P* values were determined by ANOVA or Student’s *t* test. *n* = 4–5 mice

Pin1 simultaneously activates and inactivates numerous oncoproteins and tumor suppressors, respectively^7,25^, as well as globally downregulates microRNAs in cancer cells by inhibiting their biogenesis^32^. We next assessed the extent to which ATO and/or ATRA affect protein levels of a selected subset of Pin1 substrate oncoproteins and tumor suppressors, whose protein stability is regulated by Pin1 in TNBC^25^. ATO and ATRA alone caused the dose-dependent reduction of Pin1 protein and its substrate oncoproteins, including cyclin D1^61^, NF-κB/p65^54^, β-catenin^55^, Akt^63^, c-Jun^64^, c-Myc^65^, Rab2A^34^, and caused the dose-dependent induction of Pin1 substrate tumor suppressors such as Fbw7^56^ and Smad2/3^66^ in two TNBC cell lines (Fig. 5a and Supplementary Fig. 5l). Moreover, their combination displayed cooperative effects, with the phenotypes similar to those resulting from Pin1 KO using CRISPR (Fig. 5a and Supplementary Fig. 5l). Thus, ATO and ATRA cooperately ablate Pin1 to simultaneously block multiple cancer-driving pathways.

To independently confirm the cooperative ablation of Pin1 by ATO and ATRA in TNBC cells, we performed global analyses of protein and microRNA expression after treating 231 cells with ATO and/or ATRA for 3 days. Global alterations in proteins and microRNAs in mock-treated cells were compared to the positive control Pin1 CRISPR KO 231 cells using a tandem mass tag (TMT9plex)-based proteomic approach^67^ and an NanoString nCounter microRNA Expression Assay^32^, respectively. Out of the 7003 proteins quantified across all 10 samples, 3758 proteins passed the abundance filter and were reliably quantified. Among them, 209 were altered by 1.5-fold in abundance in Pin1 CRISPR 231 cells compared with the parental WT control cells. Although ATO, ATRA, and Pin1 KO had some difference in overall expression pattern, ATO and ATRA conferred similar effects at the proteomic level, but their cooperation was obvious, with their combination most closely resembling the Pin1 KO effect (Spearman's correlation coefficient 0.69, *P* value <2.2e−16) (Fig. 5b–d and Supplementary Fig. 6a). Similarly, although ATO, ATRA, and Pin1 KO also had some different effects on individual microRNAs (Supplementary Fig. 6b), ATO and ATRA, especially in their combination, globally upregulated microRNA expression, similar to Pin1 KO (Fig. 5e). Strikingly, many of the consistently downregulated proteins across all treatments are oncogenic, and many of the consistently upregulated proteins are tumor suppressive (Supplementary Table 2). Global upregulation of microRNAs in Pin1 KO or inhibited cancer cells is also consistent with the findings that Pin1 regulates microRNA biogenesis^32,68^. Thus, multiple independent analyses demonstrate that ATO and ATRA synergistically target Pin1 to inhibit its numerous cancer-related pathways.

### ATO and ATRA cooperately inhibit Pin1 and tumor growth

Given the striking anticancer effects of ATO and ATRA in vitro, a critical question is whether they have any cooperative effects on Pin1 levels, Pin1-regulated oncogenic pathways, and tumor growth of TNBC in vivo. We thus orthotopically xenografted TNBC 231 cells into cleared mouse mammary fat pads and then treated them with ATO, ATRA, or their combination 1 week after xenograft when tumor growth was notable. Since regular ATRA has a half-life of only 45 min in humans^46^, we used 5 mg 21-day slow-releasing pellets^21^. For ATO, we used 2 mg/kg 3 times/week, a standard concentration that has widely and safely been used for treating APL in mouse models and human patients^47–49^. While ATRA and ATO alone inhibited tumor growth, their combination displayed cooperative activity, markedly inhibiting tumor growth (Fig. 5f–h).

To better recapitulate human TNBC tumors and their microenvironment, we established PDOX models for two different human TNBC tumors and treated them with ATO and/or ATRA at the same doses as above. Again, ATRA and ATO alone inhibited tumor growth, but their combination displayed cooperative antitumor activity in both PDOXs when the treatments were started after tumor growth was notable (Fig. 5i–k), or tumor volume reached 270 mm^3^ (Fig. 5l–n), or even 360 mm^3^ (Supplementary Fig. 6c–e). Notably, ATRA also induced AQP9 expression and cooperated with ATO to induce Pin1 degradation, destabilization of Pin1’s substrate oncoproteins, and stabilization of Pin1’s substrate tumor suppressors, in both TNBC cell orthotopic and PDOX tumors (Fig. 5o and Supplementary Fig. 6f). Thus, ATO and ATRA cooperatively ablate Pin1 to block multiple cancer-driving pathways and inhibit tumor growth in TNBC cell xenografts and PDOXs.

### ATO and ATRA cooperatively inhibit Pin1 and TIC self-renewal

As an independent approach to demonstrate that ATO has anticancer activity by targeting Pin1 oncogenic function and cooperating with ATRA, we chose to study TICs/CSCs of TNBCs, which are a proposed source of tumor initiation, growth, and metastasis, but are not effectively targeted by current cancer drugs^4,5^. Moreover, Pin1 is highly enriched in breast TICs and drives TIC self-renewal and tumor initiation and growth^33–35^, but whether Pin1 inhibitors would effectively target TICs is not known.

**Fig. 6:**
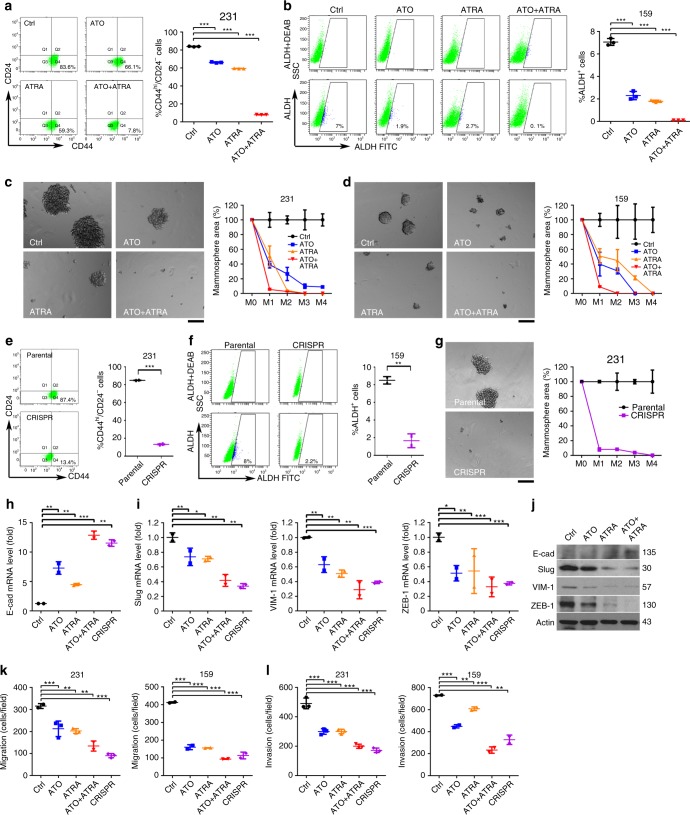
ATO and ATRA cooperatively inhibit the population and self-renewal of TICs in TNBC. **a**, **b** ATO and ATRA cooperatively reduce the population of TICs in TNBCs. Human TNBC 231 (**a**) and 159 (**b**) cells were treated with ATO (1 µM) or ATRA (10 µM) or their combination, followed by FACS analysis of the TIC-enriched CD24^−^CD44^+^ population and ALDH^+^ population. **c**, **d** ATO and ATRA cooperatively inhibit the self-renewal of TICs in TNBCs. TNBC 231 (**c**) and 159 (**d**) cells were treated with ATO (1 µM) or ATRA (10 µM) or their combination, followed by serial mammosphere formation assay to measure their TIC self-renewal. Scale bar = 150 μm. **e**–**g** Pin1 KO using CRISPR reduce the population and self-renewal of TICs in TNBCs. Pin1 CRISPR KO and control 231 cells (**e**, **g**) or 159 cells (**f**) were subjected to FACS analysis of the TIC-enriched CD24^−^CD44^+^ population (**e**) and ALDH^+^ population (**f**) or serial mammosphere formation assay (**g**). Scale bar = 150 μm. **h**–**j** ATO and ATRA cooperatively inhibit the EMT of TNBC cells. TNBC 231 cells were treated with ATO (1 µM) or ATRA (10 µM) or their combination, followed by measuring the expression of E-cadherin (**h**), and slug, vimentin, and ZEB-1 (**i**) using real-time PCR or immunoblot (**j**). **k**, **l** ATO and ATRA cooperatively inhibit migration and invasion of TNBC cells. TNBC 231 cells were treated with ATO (1 µM) or ATRA (10 µM) or their combination, followed by assaying cell migration (**k**) and invasion (**l**) using Pin1 CRISPR KO cells as controls

To examine the effects of ATO and ATRA on TICs in TNBC, we first treated 231 and 159 cells with ATO (1 µM), ATRA (10 µM), or their combination, followed by assaying the breast TIC-enriched CD24^−^CD44^+^ or ALDH^+^ population using fluorescence-activated cell sorting (FACS)^33,34^ While ATO and ATRA both significantly reduced breast TIC-enriched population, their combination cooperatively reduced the TIC population to the levels (Fig. 6a, b) close to Pin1 CRISPR cells (Fig. 6e, f). To examine the effects of ATO and ATRA on self-renewal of breast TICs, we treated TNBC cells with ATO, ATRA, or their combination, followed by a serial mammosphere formation assay. Both TNBC 231 and 159 cells formed very fast-growing spheres that did not decrease when propagated to M4 (Fig. 6c, d), indicating that mammosphere-forming cells were self-renewing at a constant rate^35^. However, after treatment with ATO or ATRA, the cells formed fewer and smaller mammospheres displaying strongly impaired mammosphere formation efficiency at M2–4. Moreover, their co-treatment displayed cooperative effects, almost completely inhibiting mammosphere formation efficiency at M1 (Fig. 6c, d), similar to Pin1 CRISPR KO (Fig. 6g). Similar results were also obtained in TNBC MDA-MB-468 cells (Supplementary Fig. 7a–d). Moreover, ATO effectively inhibited mammosphere formation efficiency at M1 in Pin1 CRISPR 231 cells expressing Pin1 or the M130I mutant, but not the M130V mutant (Supplementary Fig. 7e, f), consistent with their ATO binding (Fig. 3) Thus, Pin1 binding to ATO is required for ATO to target TICs.

Since the epithelial-to-mesenchymal transition (EMT) phenotype is another breast TIC property^69^, and is reversed by Pin1 KD or KO^33^, we also examined the effects of ATO and/or ATRA on EMT. ATO and ATRA, especially in combination, strongly induced the mesenchymal-to-epithelial transition (MET), as displayed by upregulation of epithelial markers, such as E-cadherin (Fig. 6h–j), and downregulation of mesenchymal markers, such as slug, vimentin, and ZEB-1 (Fig. 6i–j), as well as reduced cell migration and invasion equivalent to Pin1 KO using CRISPR (Fig. 6k, l and Supplementary Fig. 8a, b). Thus, ATO and ATRA cooperatively reduce the population, self-renewal, and EMT of TICs in TNBC, similar to Pin1 KO.

### ATO and ATRA cooperatively inhibit Pin1 and TIC tumor growth

**Fig. 7:**
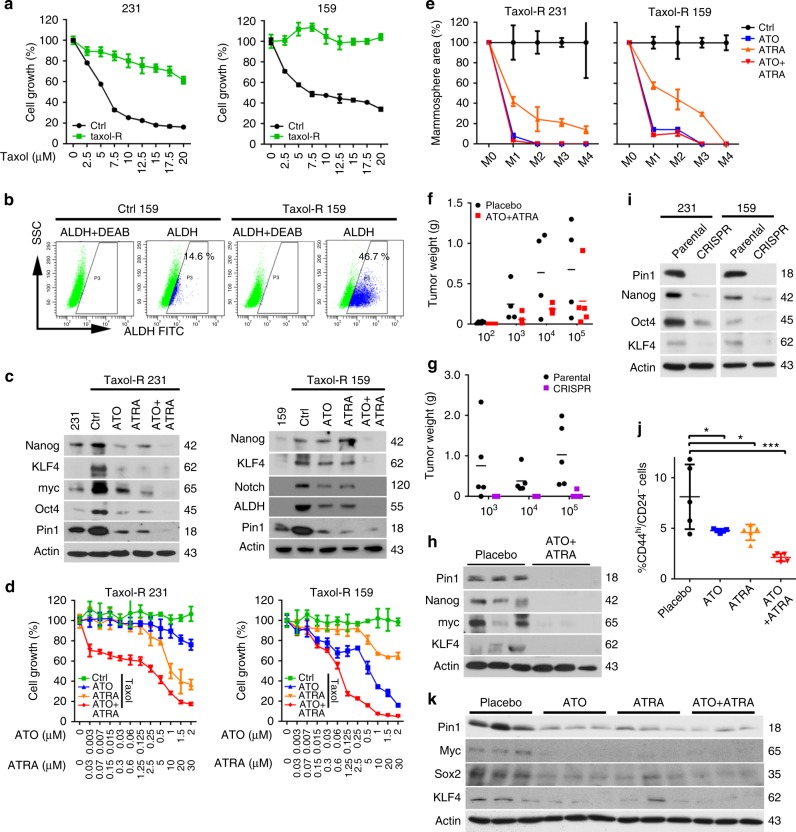
ATO and ATRA cooperatively inhibit taxol resistance, tumor initiation, and tumor growth of TICs in TNBC. **a** Generation of taxol-resistant 231 and 159 cells by treating cells with an increasing concentration of taxol over time, followed by assaying cell growth after taxol treatment. **b** Taxol-resistant 159 cells have an increased population of TICs, as assayed by ALDH using FACS analysis. **c**–**e** ATO and ATRA cooperatively reduce multiple cancer stem cell regulators, cell growth, and self-renewal of taxol-resistant TNBC cells in vitro. Taxol-resistant TNBC 231 and 159 cells were treated with ATO (1 µM) or ATRA (10 µM) or their combination, followed by measuring selected stem cells regulators using IB (**c**), cell growth (**d**), self-renewal of TICs using serial mammosphere formation assay, followed by calculating the average area of all mammospheres formed (**e**). **f**–**i** ATO and ATRA cooperatively reduce tumor initiation and growth, and CSC regulators of TNBC cells in mice similar to Pin1 KO using CRISPR. TNBC cells were treated with ATO (1 µM) and ATRA (10 µM) for 3 days, followed by being injected into subcutaneous sites of nude mice in limiting dilutions and treated with ATO (2 mg/kg, i.p., 3 times/week) and ATRA (5 mg in 21-day slow release) (**f**, **h**). Pin1 CRISPR and vector control 231 cells were used in parallel as a control (**g**, **i**). Mice were sacrificed and evaluated for tumor weight (**f**, **g**), and expression of selected CSC regulators (**h**). Pin1 CRISPR cells were analyzed for CSC regulators by immunoblot (**i**). **j**, **k** ATO and ATRA cooperatively reduce TIC population and CSC regulators in PDOXs. TNBC patient-derived tumors were transplanted into cleared mouse mammary fat pads, followed by treating mice with ATO and/or ATRA or their combination for 5 weeks. Mice were sacrificed and evaluated for the TIC population by FACS (**j**), and selected CSC regulator expression (**k**)

Breast TICs are notoriously resistant to cytotoxic chemotherapy drugs such as taxol^4,5^, commonly used to treat TNBC^70^. Since ablation of Pin1 by ATO and ATRA eliminates breast TICs, we expected that taxol-resistant TNBC cells would still be sensitive to ATO and ATRA co-treatment. To test this, we generated taxol-resistant 231 and 159 cells (Fig. 7a), followed by drug treatments. Compared with parental cells, taxol-resistant 231 and 159 cells had increased population of TICs (Fig. 7b), elevated levels of multiple CSC regulators (Fig. 7c), and increased migration and invasion (Supplementary Fig. 8a–d), as expected^4,5^. Importantly, these TIC-related phenotypes were drastically inhibited by ATO and ATRA, and particularly their combination (Fig. 7c and Supplementary Fig. 8a–d). Moreover, ATO and ATRA, especially in their combination, potently inhibited the growth of taxol-resistant cells (Fig. 7d), and also effectively inhibited self-renewal of taxol-resistant breast TICs (Fig. 7e and Supplementary Fig. 8e). Thus, ATO and ATRA combination eliminates resistance of TICs to taxol.

This raises the question of whether ATO and ATRA combination would inhibit tumor initiation and growth of breast TICs in vivo. We assayed the effects of ATO and ATRA combination therapy on tumor initiation of TNBCs using a limiting dilution assay in mice, a standard approach to determine tumor initiation^71^. Importantly, ATO and ATRA co-treatment not only effectively reduced breast TIC frequency by ~90-fold (*P* < 0.0001), but also dramatically reduced tumor growth (Fig. 7f and Table 1), similar to Pin1 KO (Fig. 7g and Table 1). Moreover, ATO and ATRA co-treatment potently downregulated multiple CSC regulators in tumors (Fig. 7h), like Pin1 KO (Fig. 7i). Finally, ATO and ATRA co-treatment also cooperatively reduced breast TIC-enriched population (Fig. 7j) and multiple CSC regulators (Fig. 7k) in PDOX tumors. Thus, ATO and ATRA cooperatively ablate Pin1 to inhibit the self-renewal, drug resistance, tumor initiation, and growth of TICs in TNBC, similar to Pin1 KO.

**Table 1:** Tumor incidence in limiting dilution assay

No. of cells injected	Tumor incidence			
	Placebo	ATO + ATRA	Parental	CRISPR
10^2^	5/6	0/6	—	—
10^3^	4/5	2/4	4/5	0/5
10^4^	5/5	4/6	5/5	0/5
10^5^	4/4	5/6	5/5	1/5
BCSC frequency	1 in 252	1 in 22,644	1 in 621	1 in 503,345
95% CI	1 in 88–1 in 722	1 in 8,220–1 in 62,382	1 in 209–1 in 1,847	1 in 71,332–1 in 3,551,801

231 xenografts from mice treated with placebo or ATO plus ATRA or parental or CRISPR 231 cells were dissociated into single-cell suspensions and injected into the flank of mice in limiting dilution. Tumor formation were observed for 6 weeks after inoculation. CSC frequency was calculated using the L-Calc software

## Discussion

ATO is approved by the FDA exclusively for the treatment of APL because it is the only leukemia that expresses the ATO presumed target PML-RARα. We have now discovered that, at clinically relevant and safe concentrations, ATO directly and noncovalently binds, inhibits, and induces degradation of Pin1, a major common regulator of cancer signaling networks, thereby inhibiting TNBC, and that these anticancer effects are abolished by disrupting ATO’s binding to Pin1. ATRA, another Pin1 inhibitor, increases cellular uptake of ATO by inducing the ATO transporter AQP9. Used together, ATO and ATRA cooperatively ablate Pin1, thereby blocking numerous cancer-driving pathways and inhibiting TICs and tumor growth of TNBC, similar to Pin1 KO in human cells and in orthotopic tumor models, including PDOX. ATO and ATRA combination not only potentiates their anticancer efficacies, but also reduces drug toxicity, which is especially important given ATO’s well-known toxicity at high doses. Thus, cooperative Pin1 inhibition by ATO and ATRA potently blocks numerous oncogenic pathways and eliminates TICs, offering a promising non-toxic approach to fighting TNBC and likely many other cancers.

A central signaling mechanism in oncogenesis is pSer/Thr-Pro^7,26^. Many oncoproteins and tumor suppressors are directly regulated by Pro-directed phosphorylation and/or trigger signaling pathways involving such phosphorylation^7,26^. Proline in a protein can exist either in the *cis* or *trans* conformation, and *cis*–*trans* conversion encounters a sufficiently high energy barrier that efficient isomerization requires catalysis by PPIases^72^. Pin1 is the only known PPIase specific to pSer/Thr-Pro motifs, which is critical as phosphorylation increases the isomerization energy barrier^52,72^. Pin1-catalyzed *cis–trans* isomerization can profoundly impact protein structure and function, as confirmed by *cis-*specific and *trans*-specific antibodies^73,74^. Since kinases, phosphatases, and proteases are *trans-*specific or *cis*-specific^7^, pSer/Thr-Pro motifs create a powerful logic gate dependent upon Pin1 for the maximal activity. Pin1 serves as a unified hub that is exploited in cancer to simultaneously turn oncoproteins on and turn tumor suppressors off^7^. Indeed, Pin1 is a master post-phosphorylation regulator of oncoproteins, tumor suppressors, and global microRNAs^7,25,32^. ATRA binds, inhibits, and induces Pin1 degradation, thereby exerting anticancer activity against APL, AML, and breast and liver cancer by blocking multiple cancer pathways^24,40–43^. Slow-releasing ATRA formulations can be used in animal studies^24,40–42^, but not in humans. Thus, there is an urgent need to develop a longer half-life ATRA formulation or Pin1-targeted ATRA derivatives, or to identify clinically usable Pin1 inhibitors.

**Fig. 8:**
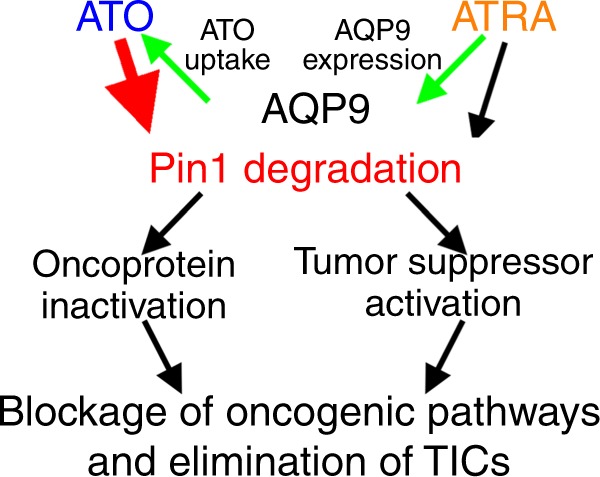
A model for the cooperation of ATO and ATRA in targeting Pin1 to block multiple oncogenic pathways and eliminate cancer stem cells, two major sources of cancer drug resistance

We have now made the unexpected discovery that ATO targets Pin1 and cooperates with ATRA to exert potent anticancer activity. Alone, ATO dose-dependently induced proteasome-dependent Pin1 degradation and inhibited cancer cell growth. Pin1 KO cells were more resistant to ATO, which was rescued by re-expressing Pin1. Thus, Pin1 inhibition contributes to ATO’s anticancer effects. ATO directly bound and inhibited Pin1 PPIase activity with an affinity of 0.1–0.2 µM, without affecting other PPIases. Importantly, ATO interacted with Pin1 active site residues, but not Cys residues, though covalent interactions with Cys have previously been proposed as the mechanism action of ATO on its targets including PML-RARα^50,51^. Furthermore, mutations of Pin1’s Cys residues had no effect on ATO binding to Pin1, whereas replacing the ATO-binding residue Met130 with Val, but not Ile, impaired ATO’s ability to bind and degrade Pin1, inhibit multiple oncogenic pathways, and inhibit TNBC cell and TIC growth in vitro and in vivo, as predicted from their co-crystal structure. Thus, noncovalent ATO binding to Pin1 is required for its ability to induce Pin1 degradation, block numerous oncogenic pathways, and inhibit TICs (Fig. 8).

The role of Pin1 in mediating ATO’s anticancer activity is further supported by the findings that human breast cancer cells were differentially susceptible to ATO and highly correlated with the rate of ATO-induced Pin1 degradation and with the expression of the ATO transporter AQP9. Importantly, AQP9 KD reduced ATO uptake and sensitivity in inducing Pin1 degradation and cell growth inhibition in ATO-sensitive cells, whereas AQP9 overexpression increased ATO uptake and reversed ATO resistance. These surprising findings led us to probe the role of ATRA, which increases AQP9 expression and enhances ATO sensitivity in APL^57,58^. Indeed, ATRA activated AQP9 promoter, increased AQP9 mRNA and protein expression, as well as enhanced ATO uptake, suggesting that ATRA may cooperate with ATO to enhance their anticancer activity. Indeed, ATO and ATRA together displayed cooperative effects leading to potent ablation of Pin1, inhibition of multiple oncogenic pathways, and inhibition of cell and tumor growth in vitro and in vivo. The synergistic effects were largely abrogated by AQP9 KD, which did not affect the ability of ATRA to reduce Pin1 and inhibit cell growth in TNBC cells. Moreover, ATO and ATRA co-treatment more potently inhibited the self-renewal, chemoresistance, and tumor initiation and growth of TICs in TNBC in vitro and in vivo. ATO and ATRA also cooperatively ablates multiple Pin1-regulated CSC regulators even in PDOXs. Significantly, these phenotypes of ATO and ATRA cooperation are similar to those resulting from Pin1 KO using CRISPR, which is also substantiated by comprehensive analyses of protein and microRNA expression. Notably, the cooperative ability of ATO and ATRA to eliminate TICs in TNBC by targeting Pin1 is consistent with the previous findings that genetic or chemical inhibition of Pin1 induces PML/RARα degradation to eradicate leukemia stem cells and treat APL without inducing myeloid differentiation^20,21,24^. Thus, ATRA cooperates with ATO to ablate Pin1 and enhance anticancer activity directly and acting indirectly on Pin1 to increase ATO uptake through AQP9 (Fig. 8). Although ATO and ATRA each have other anticancer mechanisms^16,50,51^, their cooperative Pin1 inhibition likely plays a major role in mediating their ability to block multiple cancer-driving pathways and eliminate TICs in TNBC, two major sources of drug resistance in current cancer therapy (Fig. 8).

We show that ATO directly and noncovalently binds to the common cancer signaling regulator Pin1 to block multiple cancer-driving pathways and eliminate CSCs in TNBC. These results are consistent with the previous findings that ATO shows efficacy against various hematologic malignancies and solid tumors^9,13^, given prevalent Pin1 overexpression in human cancers^6,7,25^. They are also consistent with the recent epidemiological findings that exposure to ATO-contaminated drinking water dramatically reduces overall breast cancer mortality in the affected population^15^. Moreover, we have elucidated the mechanisms underlying the striking cooperation between ATO and ATRA that gives rise to their potent anticancer effects (Fig. 8). This unique drug combination not only potently increases the efficacy of ATO, but also effectively reduces its notoriously high toxicity^9,16^. Notably, Pin1 KO in mice has no obvious defects for an extended period of time^7,37^, but prevents cancer development by overexpression of various oncogenes or loss of tumor suppressors^27–30^. Thus, ablation of Pin1 by ATO, especially when combined with longer half-life ATRA, along with AQP9 expression as a potential marker for ATO sensitivity, offers an exciting new non-toxic approach to overcome cancer drug resistance in solid tumors, as demonstrated by its safety and efficacy against APL.

In summary, our results not only reveal a novel anticancer mechanism for ATO, but also provide the first evidence that ATO, particularly in combination with ATRA, blocks multiple cancer-driving pathways and eliminates TICs in TNBC by targeting Pin1. This offers a promising, low-toxicity option for treating a broad range of cancers.

## Methods

### Cell culture and reagents

All cell lines were obtained from American Type Cell Collection (ATCC, USA). The 293T, BT549, HCC1937, HCC1806, MCF7, MDA-MB-231, MDA-MB-468, SKBR3, and T47D cells (originally obtained from ATCC and maintained in the Lu laboratory) were cultured in Dulbecco's modified Eagle's medium (DMEM) supplemented with 10% fetal bovine serum (FBS). Hs578t, NB4, and HL60 cells were cultured in Roswell Park Memorial Institute medium (RPMI) with 10% FBS. SUM159 cells were cultured in Ham’s F-12 medium with 5% FBS, insulin (5 μg/ml), and hydrocortisone (1 μg/ml). All the cells used for the experiments were tested negative for mycoplasma contamination using 4′,6-diamidino-2-phenylindole (DAPI) staining. ATO, ATRA, MG132, and cycloheximide were purchased from Sigma and ATRA-releasing pellets were from Innovative Research of America. N15-NH4Cl was purchased from Cambridge Isotope Laboratories, Inc. BME vitamins were purchased form MP Biomedicals. All Pin1 mutations, including C57A−, C57S−, C113A−, C113S−, C57AC113A−, C57SC113S−, M130V−, M130I, were generated by site-directed mutagenesis. Antibodies used against various proteins were as follows: Pin1 (1:10,000)^61^; β-actin (1:1000) from Sigma; AQP9 (G-3, 1:1000) from Santa Cruz; cyclin D1 (DCS-6, 1:1000) from BioLegend; CD44^−^APC (5599421, 1:1000) and CD24^−^PE (555428, 1:1000) from BD Biosciences; Oct-4 (ab109183, 1:1000) from Abcam; Sox2 (D6D9, 3579, 1:1000), Nanog (D73G4, 4903, 1:1000), c-Myc (D84C12, 5605, 1:1000), KLF4 antibody (4038, 1:1000), NF-κB/p65 (D14E12, 8242, 1:1000), Akt (9272, 1:1000), c-Jun (9165, 1:1000) from Cell Signaling Technology; Rab2A (15420-1-AP, 1:1000) from Proteintech Group; and FBW7 (A301-720a, 1:1000) from Bethyl Laboratories, Inc. The uncropped immunoblotting images of main figures were included in Supplementary Figures 9–12.

### Establishment of *PIN1* KO cell lines using the CRISPR/Cas9 system

Pin1 guide RNAs (gRNAs) were designed using the online CRISPR design tool (http://CRISPR.mit.edu/). The gRNA sequences were as described in Table 2^75^.

**Table 2:** The gRNA sequences of Pin1

**Name**	**Sequence of gRNA**	**PAM**
gRNA-1	AGTCACGGCGGCCCTCGTCC	TGG
gRNA-2	AGGACGAGGGCCGCCGTGAC	TGG
gRNA-3	CAGTGGTGGCAAAAACGGGC	AGG

The pLentiCRISPR construction was performed according to the protocol provided by the Zhang Lab (http://genome-engineering.org/gecko/). Oligos, (F)—5′-CACCC-gRNA and (R) AAAC-gRNA-C, were cloned into the gRNA Cloning Vector (Addgene, plasmid #49536). To obtain single clones of Pin1 KO cells, cells were transfected with the pLentiCRISPR plasmid containing each target gRNA sequence or empty vector, selected with puromycin for 3 days and isolated by colony formation assay. The single clones were validated by immunoblotting analysis and DNA sequencing.

### Inhibition of cell proliferation

Breast cancer cells were seeded at a density of 3000 cells per well in 96-well flat-bottomed plates and incubated for 72 h in culture medium. Cells were then treated with ATO, ATRA, or their combination. Control cells received dimethyl sulfoxide (DMSO) at a concentration equal to that of drug-treated cells. At 72 h, cells were counted after trypsin digestion, or medium containing 0.5 mg/ml 3-(4,5-dimethylthiazol-2-yl)-2,5-diphenyl-2*H*-tetrazolium bromide was added to each well for a 2 h incubation at 37 °C, followed by removing the media before adding 200 μl DMSO. Absorbance was determined at 570 nm. Leukemia cells were seeded at a density of 5000 cells per well in 96-well flat-bottomed plates, and incubated for 72 h in the culture medium. The number of cells was determined by CellTiter-Glo^®^ 2.0 Assay (Promega, Madison, WI, USA) following the manufacturer’s instructions.

### Generation of stable cell lines

To establish stable cell lines, cells were infected by lentivirus. For overexpression, Pin1 and Pin1 mutants were subcloned into a lentiviral vector with less optimal Kozak sequences, as described^33,34^. All shRNA constructs were purchased from Sigma-Aldrich. The target sequences of AQP9 shRNAs are GCGAACGCATTTGCAGATCAA and GCTGTGTCTTTAGCAATGTGT. To overexpress AQP9 in cells, human AQP9 cDNA was subcoloned from pEGFP-AQP9 (Plasmid #48808, Addgene) into a lentiviral vector. The 293FT cells were co-transfected with the package, envelop, and various lentivirus-expressing constructs. The virus-containing supernatant was harvested and filtered by 0.45 μm filter. For infection, the viral stock was supplemented with 8 mg/ml of polybrene.

### Intracellular arsenic concentration analysis

MDA-MB-231 treated with ATRA (10 μM), AQP9 knocked down MDA-MB-231, and AQP9 overexpressed MCF7 cells were treated with ATO (1 μM) for 0, 0.5, 1, and 2 h. Cells were collected by Cell Lifter (Corning, NY, USA) and washed with ice-cold phosphate-buffered saline (PBS) twice. Cell pellets were lysed in 0.9 ml double-distilled water by sonication for 10 min. Nitric acid was added to a final concentration of 10% to be used as an internal standard. After centrifuging at 3000 × *g* for 10 min, the supernatants were analyzed with ICP-MS as previously reported^58^.

### Evaluation of combined effects of ATO and ATRA

To evaluate the combined effect of ATO and ATRA, data were analyzed by the CalcuSyn software (Biosoft, Cambridge, UK), using the Chou–Talalay method^62^. The combination index (CI) is calculated by the formula: CI = [D]_1_/[D_*x*_]_1_ + [D]_2_/[D_*x*_]_2_. [D]_1_ and [D]_2_ are the concentrations of drug 1 and drug 2 to show a certain effect when treated with two drugs together. [D_*x*_]_1_ and [D_*x*_]_2_ are the concentrations that show the same effect with a combination of drug 1 and drug 2 when treated with each drug alone. CI values <1 indicate synergy/cooperation, whereas values >1 indicate antagonism. Synergism can be defined as follows: CI <0.1 indicate very strong synergism; 0.1–0.3 indicate strong synergism; 0.3–0.7 indicate synergism; 0.7–0.85 indicate moderate synergism; 0.85–0.90 indicate slight synergism; 0.9–1.1 indicate nearly additive effect.

### Protein stability assay

For Pin1 stability assays, cells were treated with ATO for 24 h and followed the treatment with cycloheximide (100 μg/ml) up to 36 h without ATO to block new protein synthesis, as described^24^. When cells were treated with ATO and MG132, we treated the cells with ATO for first 48 h and for following last 12 h treated them with MG132 (10 μM for MEF, 1 μM for MDA-MD-231), as described^24^. Cells were harvested at the indicated time points, and cell lysates were analyzed by immunoblotting.

### Immunoblotting analysis

Culture cells and in vivo tumor samples were lysed in RIPA buffer (50 mM Tris-HCl, pH 7.4, 150 mM NaCl, 2 mM EDTA, 1% NP40, 0.1% sodium dodecyl sulfate (SDS), 0.5% Na-deoxycholate, 50 mM NaF) containing proteinase inhibitors and then mixed with the SDS sample buffer and loaded onto a gel after boiling. The proteins were resolved by polyacrylamide gel electrophoresis and transferred to PVDF membrane. The transblotted membrane was washed twice with Tris-buffered saline containing 0.1% Tween 20 (TBST). After blocking with TBST containing 5% milk for 1 h, the membrane was incubated with the appropriate primary antibody (diluted 1:1000) in 3% bovine serum albumin (BSA)-containing TBST (Fbw7 in 5% milk) at 4 °C overnight. After incubation with the primary antibody, the membrane was washed three times with TBST for a total of 30 min followed by incubation with horseradish peroxidase-conjugated goat anti-rabbit or anti-mouse IgG (diluted 1:5000) for 1 h at room temperature. After three extensive washes with TBST for a total of 30 min, the immunoblots were visualized by enhanced chemiluminescence. Immunoblotting results were quantified using ImageJ from NIH^24^.

### PPIase assay

The PPIase activity on GST-Pin1, GST-FKBP12, or GST-cyclophilin in response to ATO were determined using the chymotrypsin-coupled PPIase activity assay with the substrate Suc-Ala-pSer-Pro-Phe-pNA, Suc-Ala-Glu-Pro-Phe-pNA, or Suc-Ala-Ala-Pro-Phe-pNA (50 mM) in buffer containing 35 mM HEPES (pH 7.8) and 0.1 mg/ml BSA at 10 °C as described^52^, with the exception that the compounds were preincubated with enzymes for 12 h at 4 °C. Ki value obtained from PPIase assay is derived from Cheng–Prusoff equation [Ki = IC50/ (1 + *S*/Km)], where Km is the Michaelis constant for the used substrate, *S* is the initial concentration of the substrate in the assay, and the IC50 value of the inhibitor, as described^24^.

### Co-crystallization, data collection, and structure determination

A near twofold excess of ATO (from a 100 mM stock) was mixed with 500 µM protein and crystallized by sitting-drop vapor diffusion at 20 °C in the following crystallization buffer: 2 M NH_4_ citrate, pH 6.5. Crystals were transferred briefly into crystallization buffer containing 25% glycerol prior to flash-freezing in liquid nitrogen. Diffraction data from complex crystals were collected at beamline 24ID of the NE-CAT at the Advanced Photon Source (Argonne National Laboratory). Data sets were integrated and scaled using XDS^76^. Structures were solved by molecular replacement using the program Phaser^77^. The ligand was positioned manually and refined using Buster and Rhofit. Iterative manual model building and refinement using Phenix and Coot led to a model with excellent statistics (Supplementary Table 1). The ATO-Pin1 co-crystal structure is being deposited into the NCBI Database (PDB ID is 6DUN).

### NMR analysis

Uniformly ^15^N-labeled PIN1 catalytic domain covering residues 51–163 (77KQ, 82KQ) was prepared at 100 μM in 50 mM sodium phosphate/100 mM sodium sulfate pH 6.6 buffer which contained 5 mM EDTA, 2 mM dithiothreitol, and 10% D_2_O; ATO was added to 0×, 1×, 2.5×, or 5× final concentration relative to protein. Standard methods were used to acquire ^1^H-^15^N HSQC spectra at 25 °C on a Bruker 500 MHz NMR spectrometer equipped with a BBO probe, using 2048 (^1^H) x 256 points (^15^N) and 32 scans per increment (total time approximately 3 h per experiment), linear-predicted 1× in the indirect dimension, and zero-filled to a final 2048 × 1024 dataset. Data were processed in Topspin (Bruker) and analyzed using CcpNmr analysis^78^. The weighted average chemical shift difference was calculated as $\Delta = \sqrt {1/2 \ast ((\Delta H)^2 + (\Delta N/5)^2)}$, where Δ*H*/Δ*N* is the change in p.p.m. of ^1^H or ^15^N for the indicated crosspeak. The significance threshold for the chemical shift changes was calculated based on the average chemical shift across all residues plus the standard deviation, in accordance with standard methods^79^.

### Nanostring microRNA profiling

After 3 days’ treatments with DMSO, ATO, and/or ATRA, cell pellets were collected, along with Pin1 CRISPR KO cells, followed by isolating total RNA using miRNeasy Mini Kit (Qiagen, Germany) according to the manufacturer's instruction, as described^32^. Expression profiling of global microRNAs in these samples was determined by Dana-Farber Cancer Institute Molecular Biology Core Facilities using NanoString nCounter microRNA Expression Assays, followed by data analysis using the NanoString nCounter software^32^. Dot plots were created using GraphPad PRISM7.0a (GraphPad Software, Inc., USA). The NanoString data have been deposited in NCBI’s Gene Expression Omnibus and are accessible through GEO Series accession number GSE116264.

### Quantitative protein analysis using multiplex quantitative proteomic analysis

After 3 days’ treatments with DMSO, ATO and/or ATRA, cell pellets were collected, along with Pin1 CRISPR KO cells. Expression profiling of global proteins in these samples was determined by the Thermo Fisher Scientific Center at Harvard Medical School for Multiplexed Proteomics (TCMP@HMS), using a tandem mass tag-based approach, as described^67^. Each protein was normalized to the summed amount of quantified proteins within the sample, and changes of the relative proteins abundance were computed by normalizing each treatment to the untreated control. Proteins with <0.01% abundance were filtered out given their high variance. A second threshold of 1.5-fold change was chosen to focus on the proteins with largest alterations, as previously described^67^. Heatmaps showing relative protein abundance changes were generated using GENE-E (3.0.215). Correlation plots and correlation matrix heatmap were created by customized R script using reshape2, LSD, and ggplot2 packages. The RAW format data have been deposited in the ProteomeXchange via the PRIDE partner repository, with dataset identifier PXD010224. The identified proteins and peptides are included in Supplementary Data 1. Both unnormalized and processed data are included.

### ATO doses

The doses used for ATO in our in vitro and in vivo studies have been widely used for previous studies on APL cells and APL mice^47–49^. These doses are clinically relevant and safe in treating APL patients. The current dosing recommendation for ATO in APL patients is 0.15 mg/kg per day. According to the FDA guidelines, to convert mouse dose in mg/kg to human equivalent doses in mg/kg, either divide mouse dose by 12.3 or multiply mouse dose by 0.08^80^. Therefore, we treated mice with 2 mg/kg/ intraperitoneally (i.p.), 3 times/week. From a phase 1 trial and pharmacokinetic study of ATO in children and adolescences, at 0.15 mg/kg per day, the median (range) plasma arsenic maximum concentration (*C*_max_) is 0.28 μM (0.11–0.37 μM); area under the plasma concentration time curve (AUC_0–24_) is 2.5 μM-h (1.28–3.85 μM-h)^81^. According to this study, we treated cells with a range from 0.125 to 2 μM ATO. In this range, the max dose is closed to the average of AUC_0–24_.

### Flow cytometric analysis

To assess cell surface expression of CD44 and CD24, cells were washed with PBS, harvested by non-enzymatic cell dissociation solution, and resuspended in blocking solution (Ca^2+^, Mg^2+^-free PBS containing 1% fetal calf serum (FCS)). Cells were then incubated with antibodies for 20 min at 4 °C, washed with PBS, and labeled with secondary antibody for 30 min at 4 °C. Cells were washed and analyzed on a BD LSRII cytometer. To assess high ALDH activity, it was performed according to the manufacturer’s guidelines (STEMCELL Technologies). CSC populations were identified as CD44^hi^/CD24^−^ in MDA-MB-231 and MA-MB-468 cells and as ALDH^+^ in SUM159 cells.

### Mammosphere formation

Single-cell suspensions were plated on ultra-low attachment plates (Corning), at a density of 500 cells per well in 3:1 serum-free DMEM/MammoCult medium (STEMCELL Technologies) with 0.8% methylcellulose (Sigma). After 8–10 days in culture, mammospheres were collected by centrifugation and dissociated enzymatically (5 min in 1:1 TrypLE/DMEM at 37°C) and mechanically by passing through 26 G needles. Single cells were counted and replated at a density of 500 cells per well for subsequent passages. We took entire images of mammosphere-culturing wells and then calculated the total area and number of all mammospheres formed using ImageJ, followed by calculating the average area of all mammospheres as described^82^.

### Cell migration and invasion

For migration assay, the underside of Transwell (Millipore) polycarbonate membrane was coated with fibronectin. Cells resuspended in 10% FCS medium were plated onto the upper chamber, and the medium containing 20% FCS was added to the lower chamber. Cells were incubated at 37 °C for several hours. At the endpoint of incubation, cells that had migrated to the lower membrane surface were fixed by 4% formaldehyde and stained with DAPI for counting. For invasion assay, the Transwell membrane was coated with Geltrex (Invitrogen).

### Animal studies

For xenograft experiments, 5 × 10^5^ of MDA-MB-231 cells or Pin1 CRISPR 231 cells stably re-expressing Pin1 or its mutants were injected orthotopically into the cleared mammary fat pads of 8-week-old NOD.Cg-*prkdc*^*scid*^*ll2rg*^*tm1Wjl*^*/Szj* (termed NSG) mice (Jackson Laboratories). One week later, tumor growth was just about notable by sight, mice were randomly selected to receive treatments with ATO (2 mg/kg, i.p., 3 times/week, Sigma) and/or subcutaneous implantation of 5 mg 21-day ATRA-releasing pellets (Innovative Research of America) or placebo. For limiting dilution xenograft experiments, cells were treated with ATO (1 µM) and ATRA (10 µM) for 3 days and injected subcutaneously into flank of 8-week-old BALB/c nude mice (Jackson Laboratories) and continuously treated with ATO (2 mg/kg, i.p., 3 times/week) and 5 mg 21 day ATRA-releasing pellets. Two patient-derived models of human breast cancer (model ID: TM00089 and TM00096) were purchased from Jackson Laboratories. Tumors were diced to 4 × 2 × 1 mm^3^ sized fragments and implanted into the mammary fat pads of NSG mice as previously reported^83^. When PDX tumors reached to the size as described in the text, mice were randomly selected to receive treatments. Tumor sizes were measured by a caliper and tumor volumes were calculated using the formula *L* x *W*^2^ × 0.52, where *L* and *W* represent length and width, respectively. All animal experiments were approved by the IACUC of the Beth Israel Deaconess Medical Center, Boston, MA, USA.

### Statistical analysis

Experiments were routinely repeated at least three times, and the repeat number was increased according to effect size or sample variation. We estimated the sample size considering the variation and mean of the samples. No statistical method was used to predetermine sample size. No animals or samples were excluded from any analysis. Animals were randomly assigned groups for in vivo studies; no formal randomization method was applied when assigning animals for treatment. Group allocation and outcome assessment was not done in a blinded manner, including for animal studies. Limiting dilution data were analyzed by the single-hit Poisson model using a complementary log–log generalized linear model with the L-Calc Software (STEMCELL Technologies). All data are presented as the means ± SD, followed by determining significant differences using the two-tailed Student's *t* test or analysis of variance (ANOVA) test, where *P < 0.05, **P < 0.01, and ***P < 0.001.

### Data availability

The authors declare that the main data supporting the findings of this study are within the article and its Supplementary Information files. Extra data are obtained from the corresponding authors upon request.

## Electronic supplementary material

Supplementary Information

Supplementary Data 1
