# Supplementary material for: Arsenic targets Pin1 and cooperates with retinoic acid to inhibit cancer-driving pathways and tumor-initiating cells
Source: Nat Commun. 2018 Aug 9;9:3069. doi: 10.1038/s41467-018-05402-2 (PMC6085299; doi:10.1038/s41467-018-05402-2)
Supplement: Supplementary file 1 — Supplementary Information [file 41467_2018_5402_MOESM1_ESM.pdf]

Supplementary Information

Arsenic targets Pin1 and cooperates with retinoic acid to inhibit cancer-driving pathways and tumor-initiating cells

Kozono & Lin et al.

Supplementary Figure 1

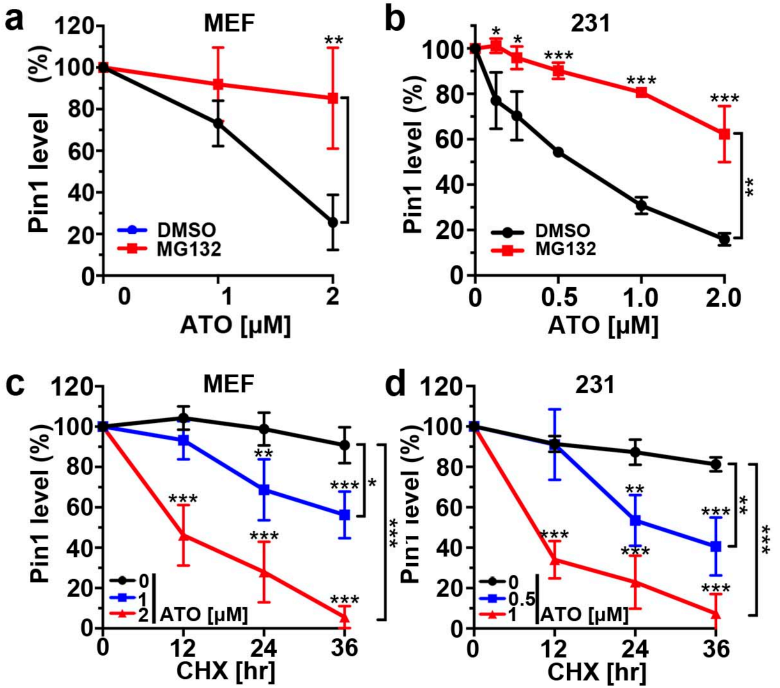

Supplementary Figure 1. The quantification data of ATO-induced Pin1 degradation.

(a, b) ATO-induced Pin1 downregulation is rescued by proteasome inhibition in MEFs **(a)** and 231 cells **(b)**. MEFs or 231 cells were treated with different concentrations of ATO in the absence or presence of MG132, followed by quantification.

(c, d) ATO dose-dependently reduces Pin1 protein stability in MEFs **(c)** and 231 cells **(d)**. MEFs or 231 cells were treated with different concentrations of ATO in the absence or presence of cycloheximide, followed by quantification.

Supplementary Figure 2

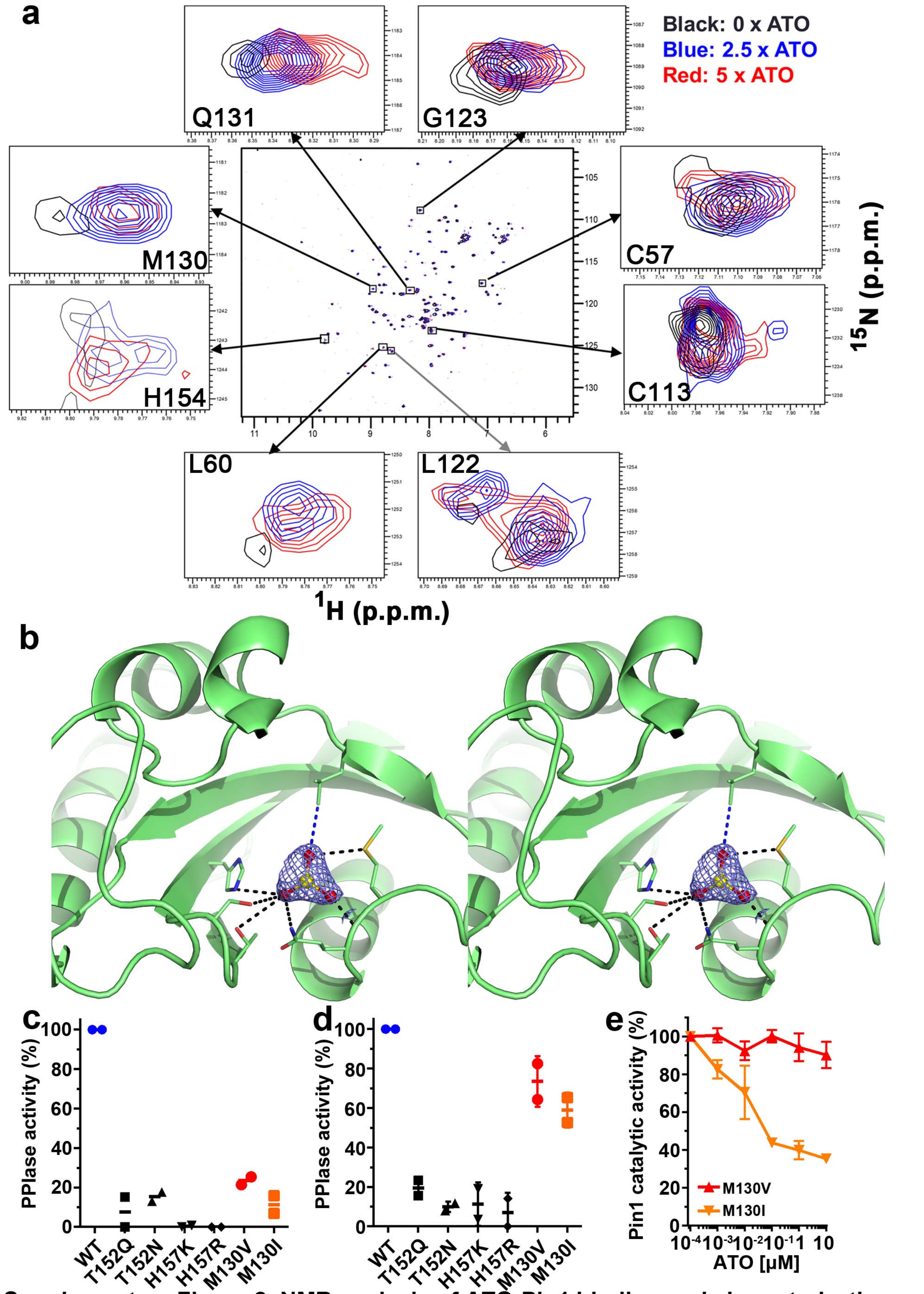

Supplementary Figure 2. NMR analysis of ATO-Pin1 binding and characterization of PPIase activity of Pin1 mutants.
(**a**) Upon addition of 2.5 or 5 x ATO to Pin1, total six residues in Pin1 show significant chemical shift changes at both ATO concentrations, with two Cys also being highlighted.
(b) A stereo image of a portion of the ATO electron density map in the Pin1-ATO crystal.
(**c, d**) 50 ng (c) and 125 ng (d) of Pin1 and its mutants were subjected to the standard chymotrypsin-coupled PPIase assay.
(**e**) 125 ng of Pin1 M130V and M130I mutants were incubated with various concentrations of ATO, followed by chymotrypsin-coupled PPIase assay.

Supplementary Figure 3

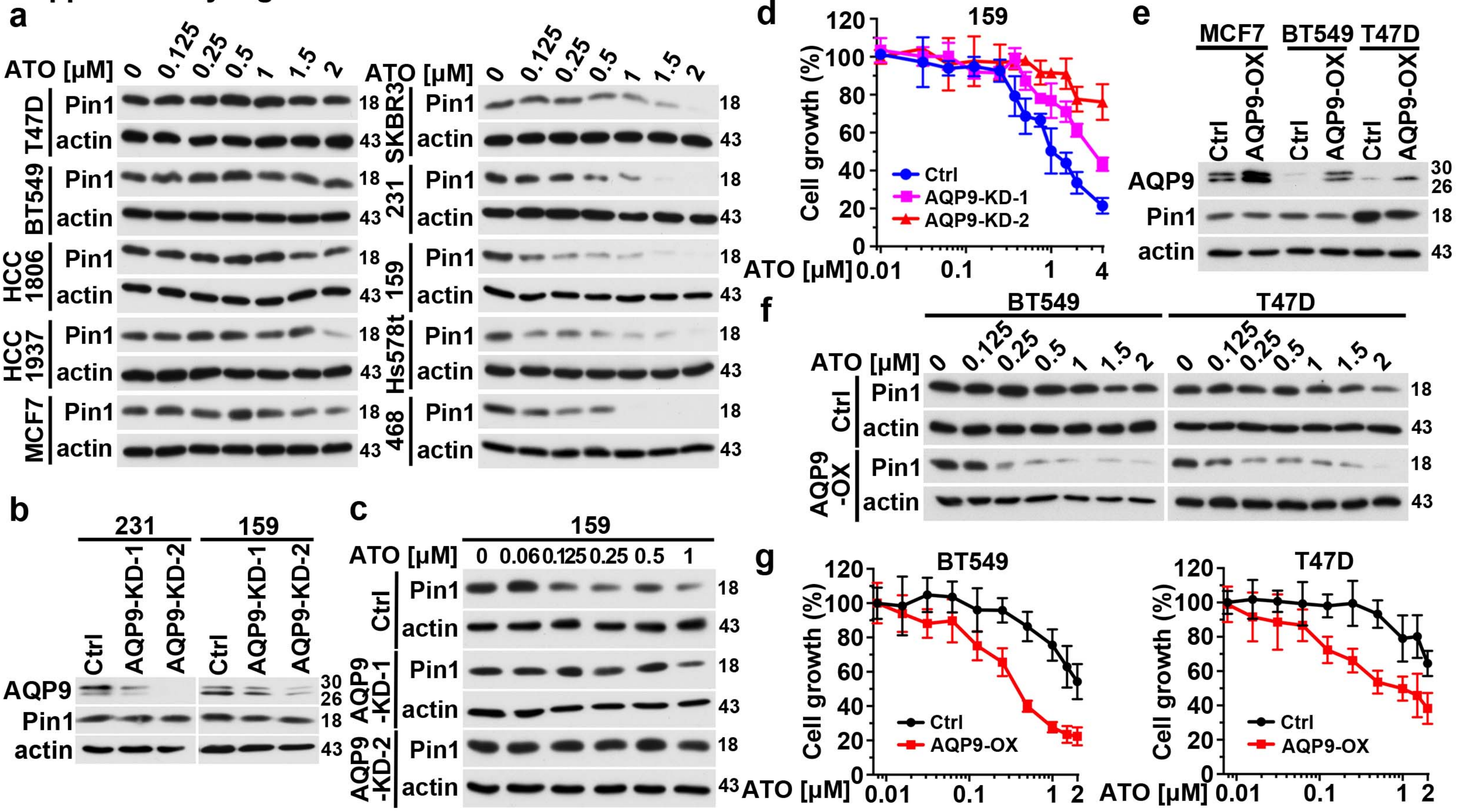

Supplementary Figure 3. Effects of ATO on Pin1 protein levels in various human breast cancer cells and AQP9 knockdown abolishes the ability of ATO to degrade Pin1 and inhibit cell growth.
(**a**) The ability of ATO to induce Pin1 degradation in breast cancer cells. 10 human breast cancer cells were treated with different concentrations of ATO for 3 days, followed by assaying Pin1 protein levels by immunoblot.
(**b-d**) AQP9 KD reduces ATO sensitivity. Stable AQP9 knockdown in 159 cells using two related shRNA lentiviral vectors were treated with different concentrations of ATO for 3 days, followed by assaying AQP9 levels **(b)**, Pin1 levels **(c)**, and cell viability **(d)**.
(f, g) AQP9 OX reverses ATO resistance. Stable AQP9 OX in three ATO-resistant cells were treated with different concentrations of ATO for 3 days, followed by assaying AQP9 and Pin1 levels **(f),** and cell viability **(g)**.

Supplementary Figure 4

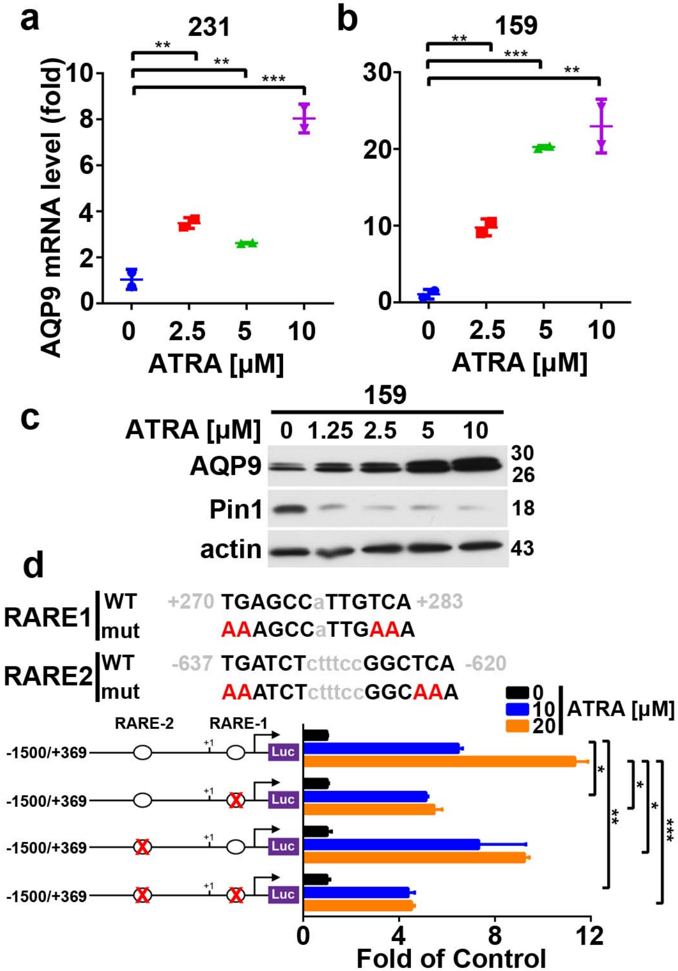

Supplementary Figure 4. ATRA induces AQP9 promoter, mRNA and protein in TNBC cells.

(**a, b**) ATRA induces transcription of AQP9 in TNBC cells. RT-PCR analysis of AQP9 induction in 231 **(a)** and 159 cells **(b)** treated with different concentrations of ATRA for 7 days.

(**c**) ATRA induces AQP9 protein expression in TNBC cells. Immunoblotting analysis of AQP9 protein upregulation in 159 cells treated with different concentrations of ATRA for 7 days.

(**d**) ATRA increases AQP9 promoter activity. The predicted sequence of RARE within the 1.5 kb promoter of AQP9 and the sequences of AQP9 promoter RARE-binding mutants used in this study (top). 231 cells transfected with indicated promoter-driven reporter constructs were treated with ATRA for 3 day, followed by assaying luciferase activity using Dual-Luciferase Reporter Assay System (Promega). All numerical data are mean ± S.D. *, $p<0.05$; **, $p<0.001$; ***, $p<0.0005$, n=3.

Supplementary Figure 5

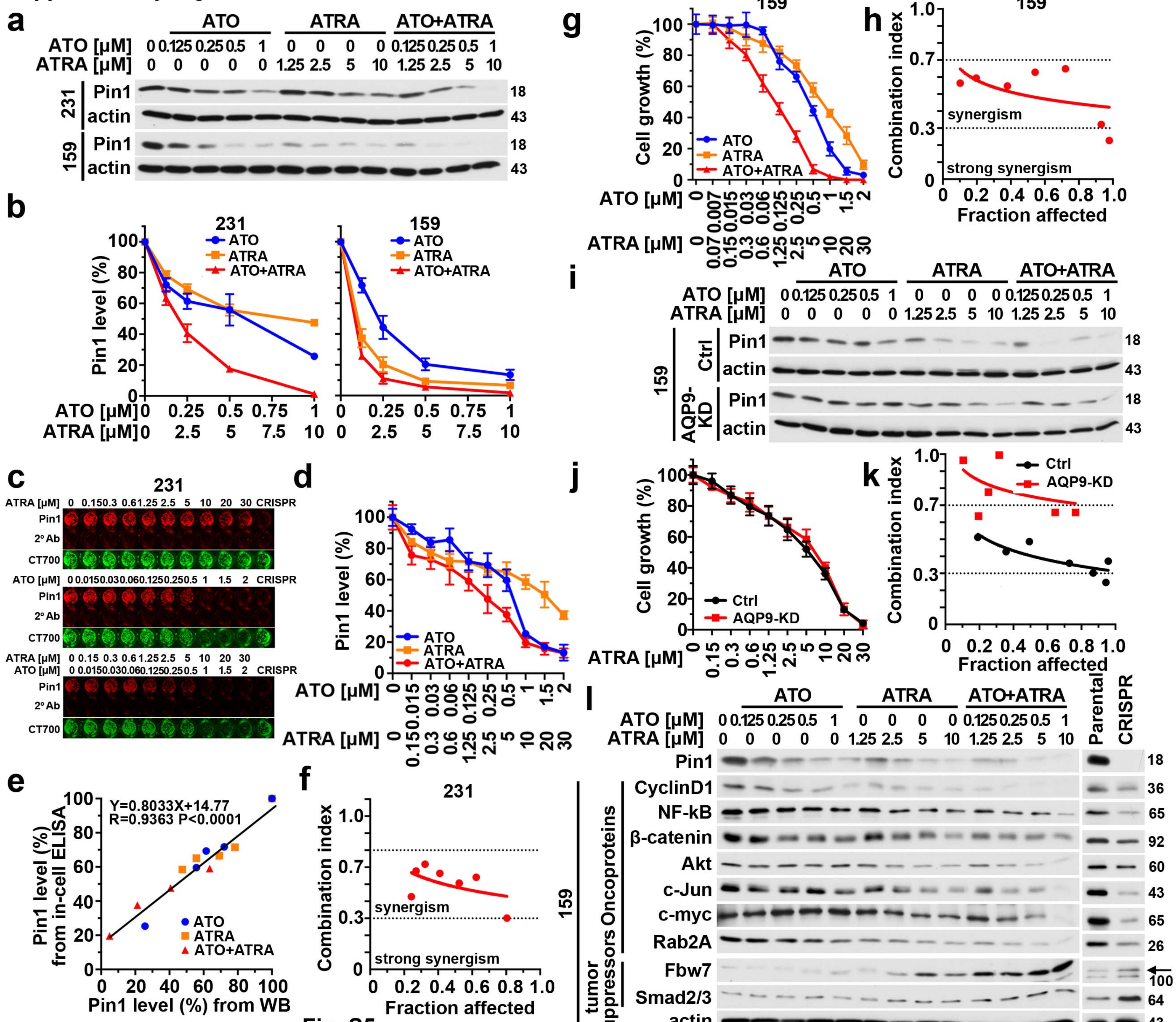

Fig. S5

Supplementary Figure 5. ATO and ATRA synergistically inhibit cell growth and induce Pin1 degradation with decreasing multiple oncoproteins and inducing tumor suppressor proteins in TNBC cells.

(**a, b**) ATO synergizes with ATRA to induce Pin1 degradation in TNBC cells. 231 and 159 cells were treated with ATO and/or ATRA for 72 hr, followed by analyzing Pin1 levels using immunoblotting.

(**c-f**), 231 cells were treated with different concentrations of ATO or ATRA or their combination for 72 hr, followed by assaying Pin1 levels using in-cell ELISA **(c, d)**, evaluating their correlation with Pin1 levels determined by immunoblotting **(e)**, and calculating their synergistic effects using CalcuSyn software **(f)**. Pin1 CRISPR cells were used as a negative control in in-cell ELISA.

(**g, h**) ATO synergizes with ATRA to inhibit cell growth in 159 cells. 159 cells were treated with ATO and/or ATRA for 72 hr, followed by counting cell number **(g)**, and determining their synergy using CalcuSyn software **(h)**.

(**i-k**), AQP9 KD abolishes ATRA synergy with ATO, but does not affect ATRA sensitivity. Control or AQP9 KD 159 cells were treated with different concentrations of ATO, or ATRA or their combination for 72 hr, followed by assaying Pin1 protein levels using immunoblot **(i)** and cell viability **(j)**, followed by determining their synergy using CalcuSyn software **(k)**.

(**l**) ATO and ATRA synergistically turn off many oncoproteins and turn on many tumor suppressors in TNBC cells in vitro, similar to Pin1 KO using CRISPR. Human 159 cells were treated with different concentrations of ATO or ATRA or their combination for 72 hr, followed by IB for detecting different proteins. Pin1 CRISPR KO and control 159 cells were used as controls. The results are expressed as mean ± S.D. and the p values determined by Student's t-test.

Supplementary Figure 6

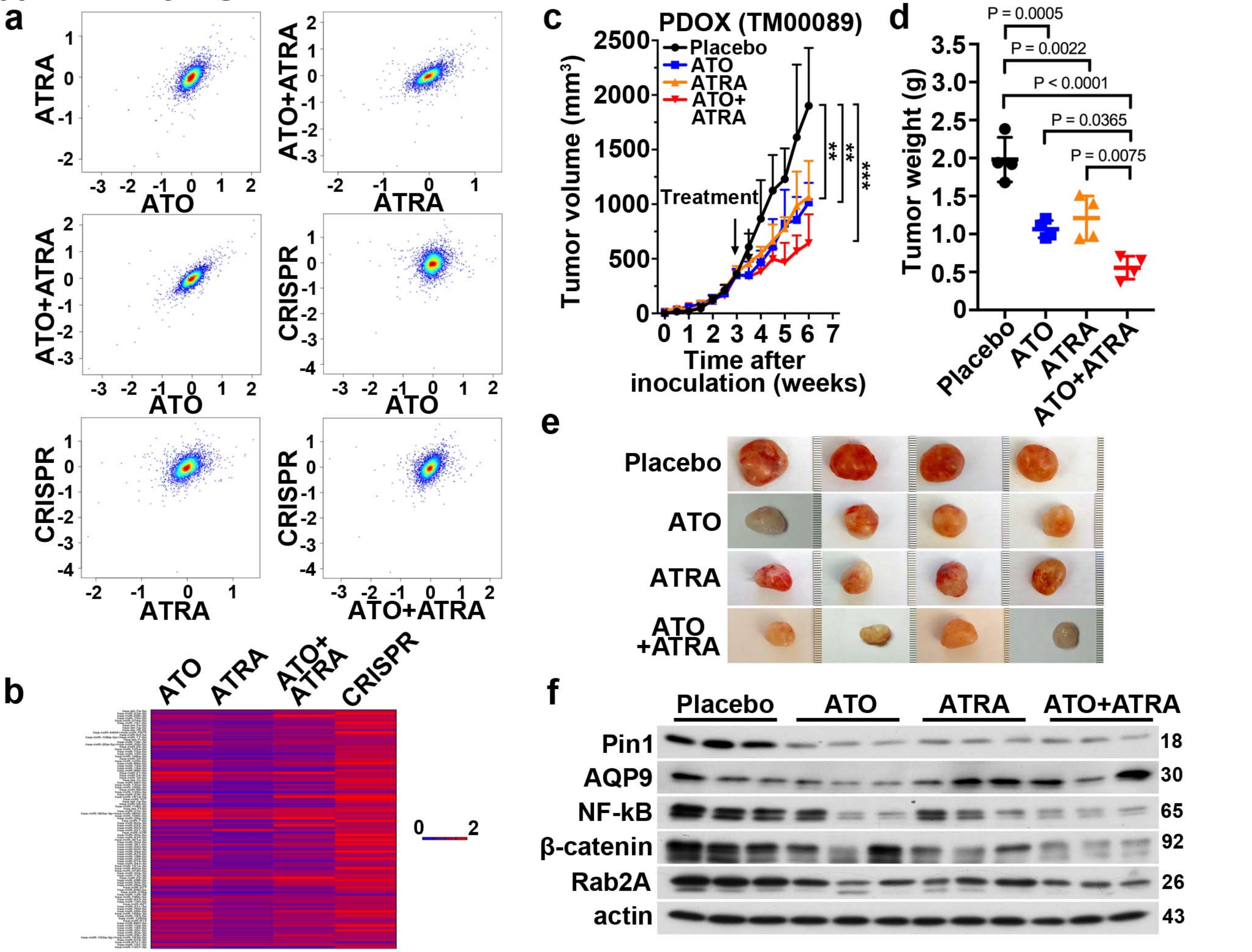

Supplementary Figure 6. The synergistic proteomic effects and in vivo tumor inhibition of ATO and ATRA.

(**a**) ATO and ATRA synergistically induced proteomic profile changes consistent with Pin1 CRISPR KO. 231 cells were treated with DMSO, 1 μM ATO, 10 μM ATRA or combination for 72 hr, followed by protein preparation for quantitative mass spectrometry analyses.
231 Pin1 CRISPR KO was added as a positive control. 3758 proteins that passed the abundance filter were subjected to xyplots for visualizing the correlation.
(**b**) ATO and ATRA globally upregulates microRNA expression in TNBC cells like Pin1 CRSIPR KO. The transformed ratio of treated versus control was used to generate the heatmap, with color slider showed up.
(**c-f**) ATO and ATRA synergistically inhibit tumor growth and multiple oncogenic pathways in the PDOX mouse model. Female NSG mice were orthotopically inoculated into mammary fat pads with patient-derived TNBC cells (TM00089, Jackson Laboratory). After tumors reached to 360 mm3, mice were treated with ATO (2mg/kg, ip, 3 times/week) or ATRA (5 mg 21 day slow-releasing pellet implant) or their combination. Tumor sizes were weekly measured **(c)** and mice were sacrificed after 3 weeks to collect tumor tissues **(e)** and measure their weights **(d)**, as well as their expression of AQP9, Pin1 and selected Pin1 substrates by IB **(f)**. The results are expressed as mean ± S.D. and the p values determined by Student's t-test.
n=4 mice

Supplementary Figure 7

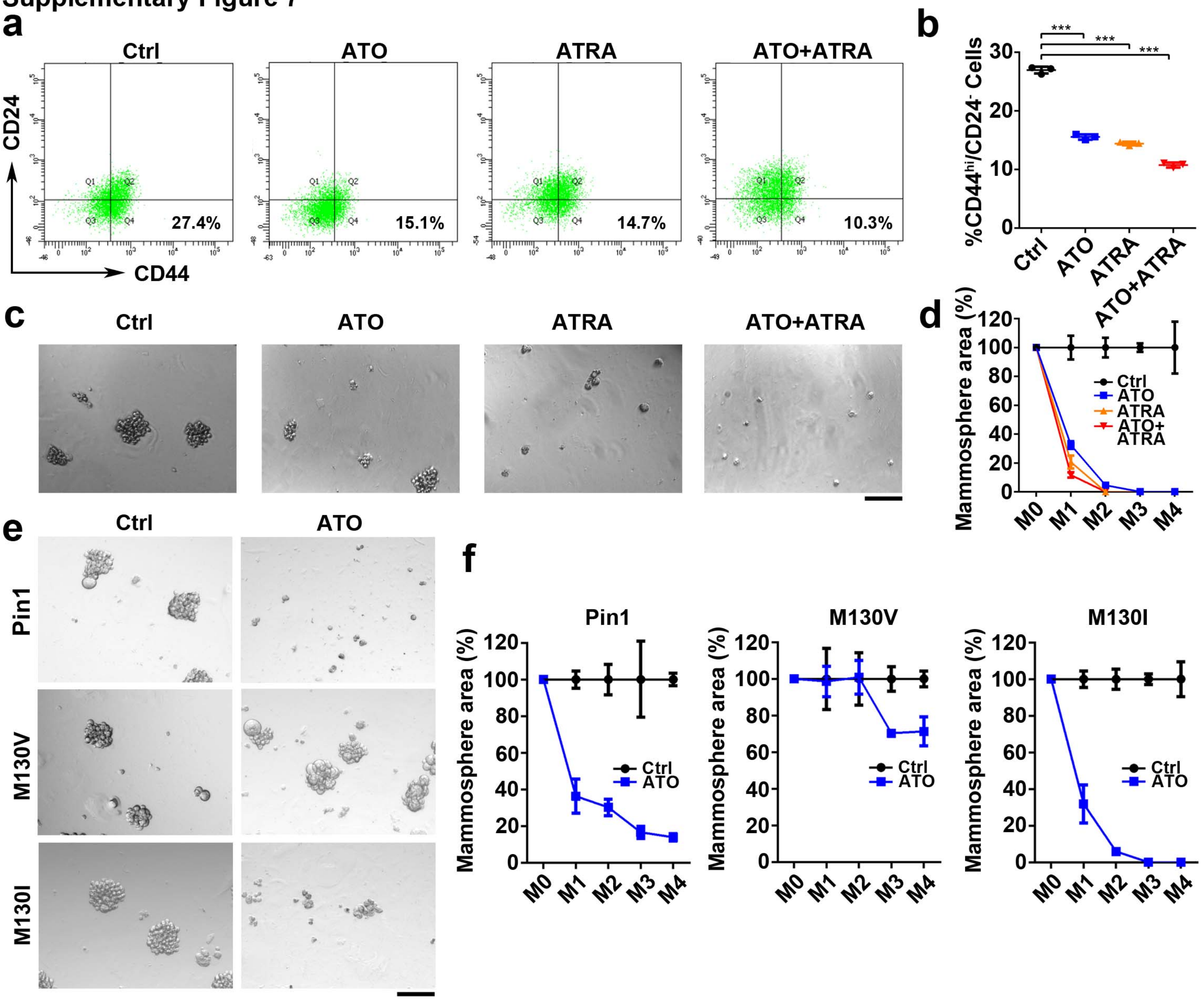

Supplementary Figure 7. ATO and ATRA synergistically reduce the population and self-renewal of TICs in TNBC 468 cells, and Pin1 M130V, but not M130I mutation, is resistant to ATO-induced inhibition of TIC self-renewal.

(**a, b**) 468 cells were treated with ATO or ATRA or their combination, followed by assaying breast TIC-enriched CD24-CD44+ population using FACS analysis.

(**c, d**) 468 cells were subjected to a serial mammosphere formation assay in the absence or presence of ATO or ATRA or their combination. Scale Bar = 150 μm.

(**e, f**) Pin1 CRISPR 231 cells stably expressing Pin1 or its mutants were treated with ATO or vehicle, followed by measuring their TIC self-renewal activity in the presence or absence of ATO using the serial mammosphere formation assay. Scale Bar = 150 μm.

Supplementary Figure 8

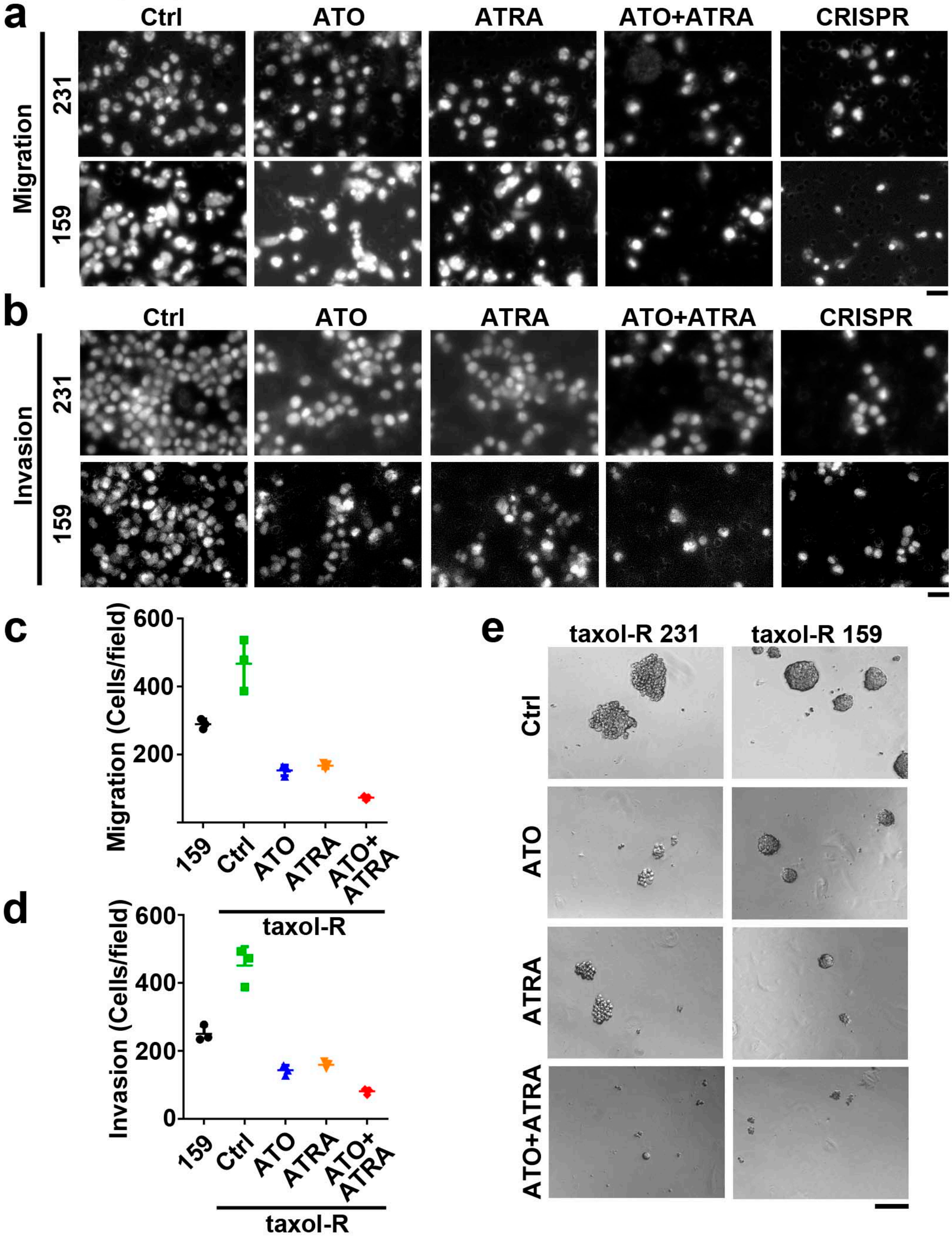

Supplementary Figure 8. ATO and ATRA synergistically inhibit the EMT phenotypes of TNBC cells and reduce migration and invasion and TIC self-renewal of taxol-resistant TNBC cells.

(**a, b**) 231 and 159 cells were treated with ATO or ATRA or their combination, followed by assaying the migration **(a)** and invasion **(b)** phenotypes, with Pin1 KO cells as controls. Scale Bar = 10 μm.

(**c, d**) Taxol-resistant 159 cells were treated with ATO or ATRA or their combination, followed by assaying their migration **(c)** and invasion **(d)**.

(**e**) Taxol-resistant 231 and 159 cells were subjected to mammosphere forming assay in the absence or presence of ATO or ATRA or their combination. Scale Bar = 150 μm.

Supplementary Figure 9

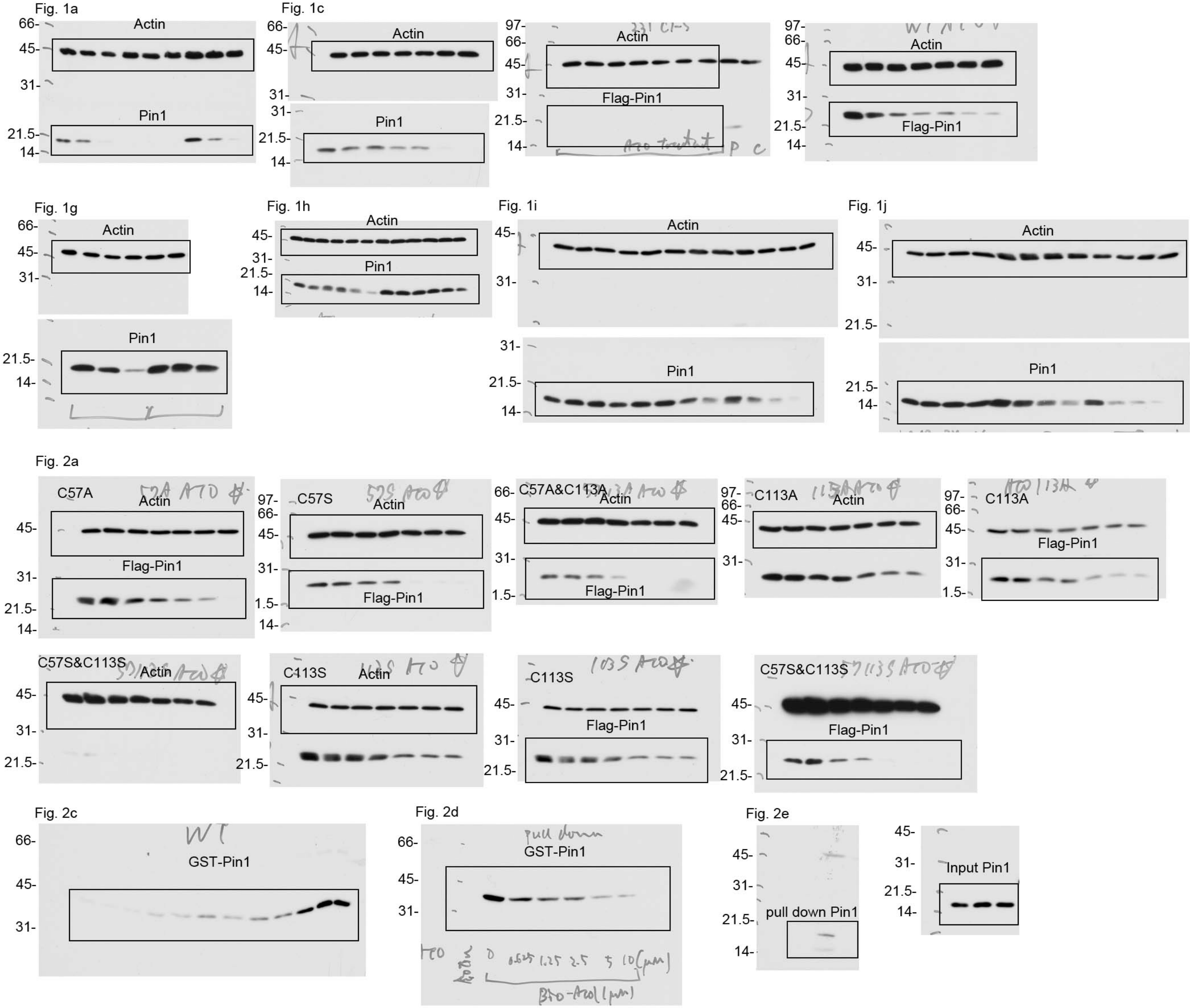

Supplementary Figure 9. Uncropped images of western blots experiments of Figure 1 and Figure 2. Molecular weight markers are indicated. Rectangles represent the cropped images shown in figures.

Supplementary Figure 10

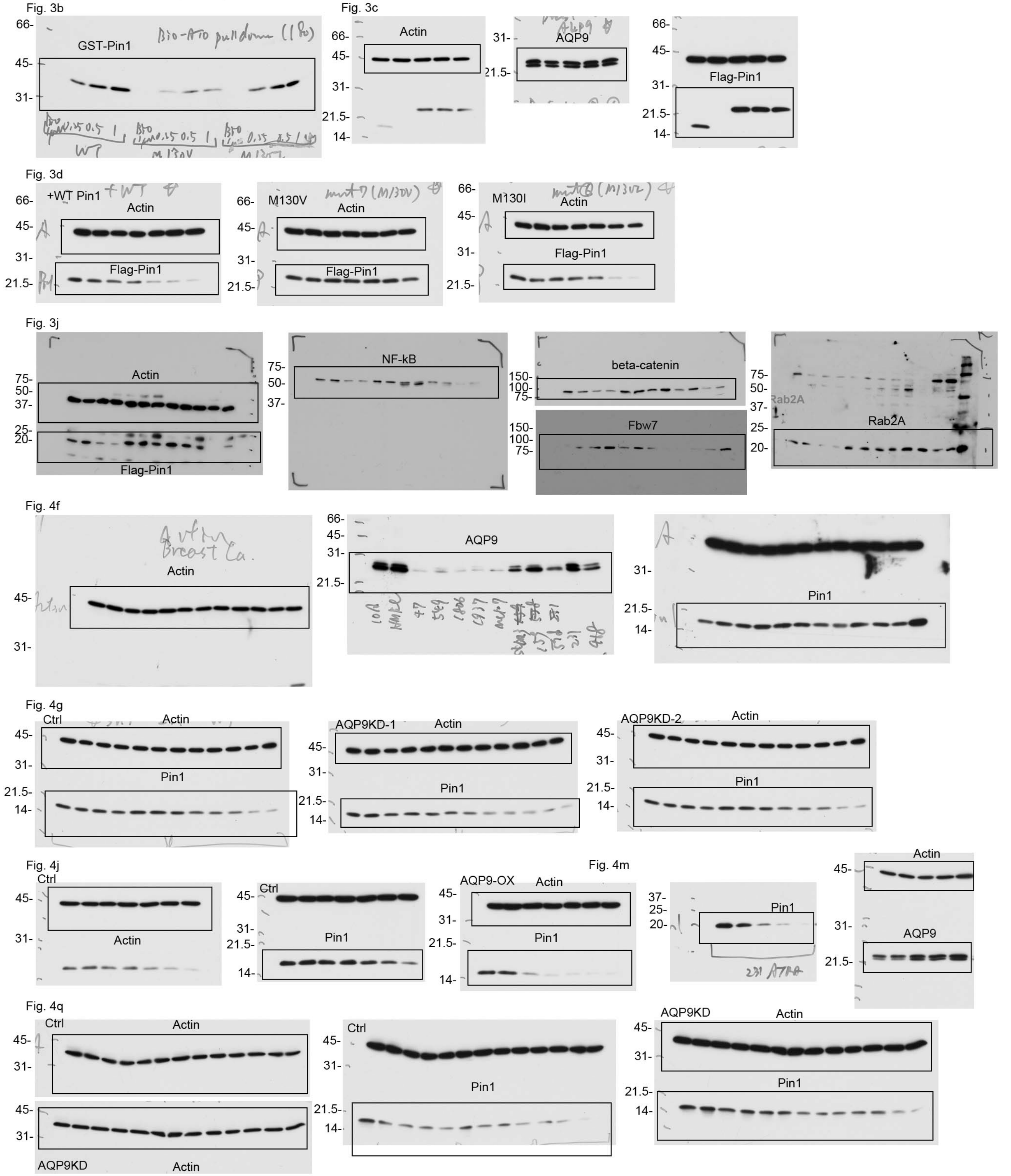

Supplementary Figure 10. Uncropped images of western blots experiments of Figure 3 and Figure 4. Molecular weight markers are indicated. Rectangles represent the cropped images shown in figures.

Supplementary Figure 11

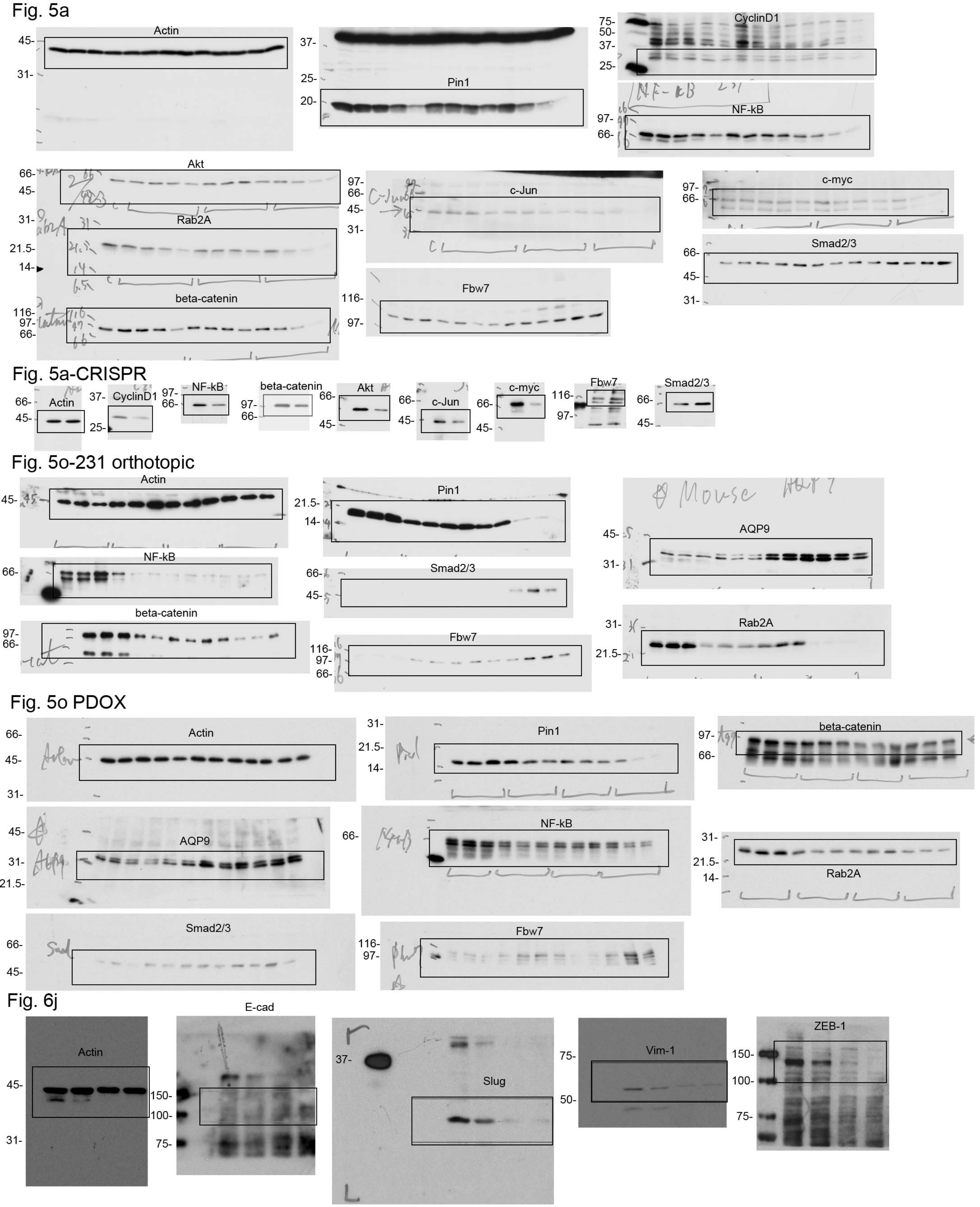

Supplementary Figure 11. Uncropped images of western blots experiments of Figure 5 and Figure 6. Molecular weight markers are indicated. Rectangles represent the cropped images shown in figures.

Supplementary Figure 12

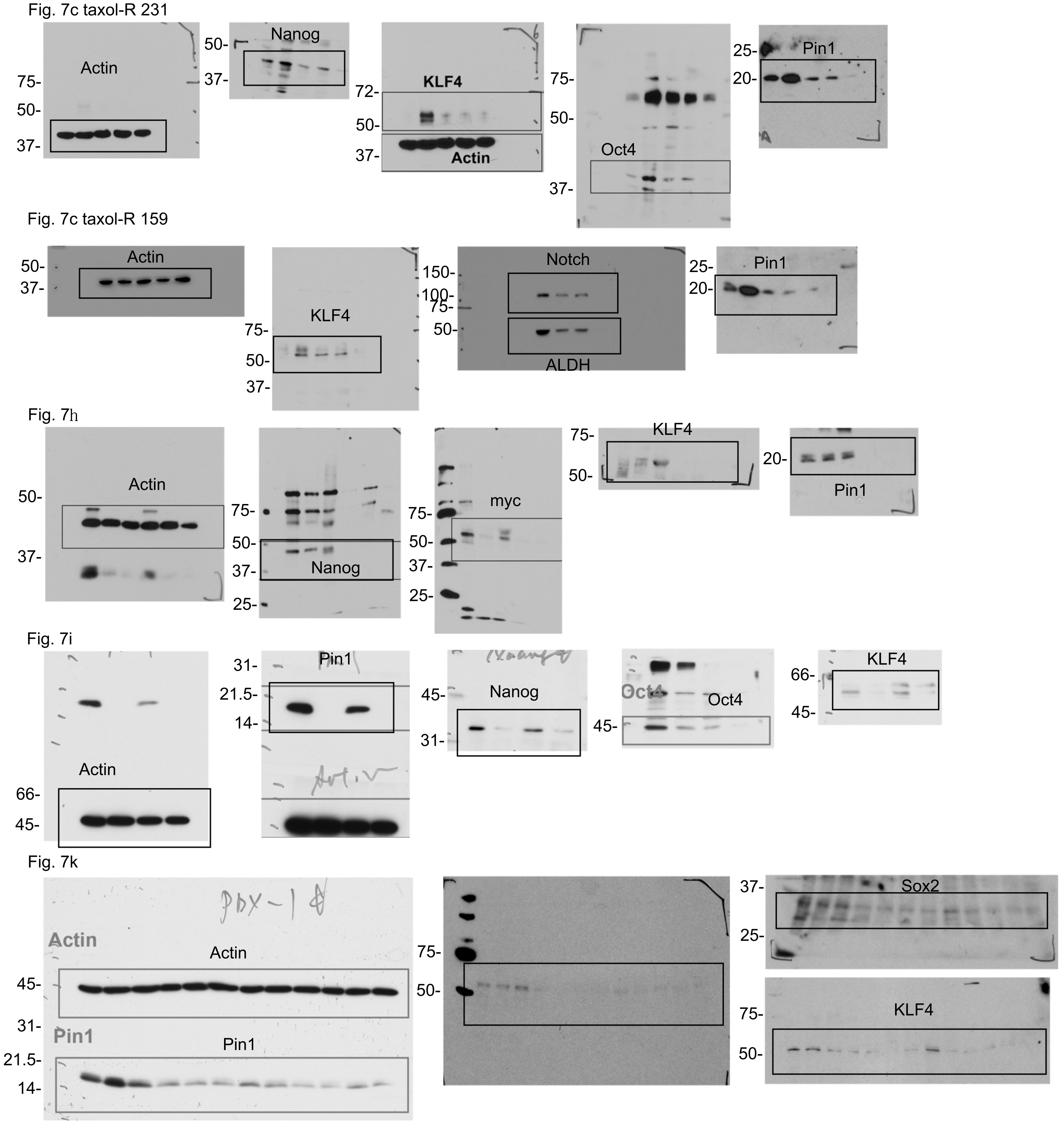

Supplementary Figure 12 Uncropped images of western blots experiments of Figure 7. Molecular weight markers are indicated. Rectangles represent the cropped images shown in figures.

Supplementary Table 1. Data collection and refinement statistics of the Pin1-ATO co-crystal structure

Wavelength	0.9792
Resolution range	50.68 - 1.59 (1.647 - 1.59)
Space group	C 1 2 1
Unit cell	117.27 36.21 51.59 90 100.75 90
Total reflections	86720 (7790)
Unique reflections	28643 (2849)
Multiplicity	3.0 (2.7)
Completeness (%)	0.99 (0.99)
Mean I/sigma(I)	8.93 (1.08)
Wilson B-factor	17.80
R-merge	0.08055 (1.019)
R-meas	0.09824 (1.271)
CC1/2	0.997 (0.363)
CC*	0.999 (0.73)
Reflections used in refinement	28639 (2845)
Reflections used for R-free	1457 (154)
R-work	0.1521 (0.2783)
R-free	0.1987 (0.3558)
CC(work)	0.962 (0.745)
CC(free)	0.943 (0.582)
Number of non-hydrogen atoms	1929
macromolecules	1767
ligands	8
Protein residues	229
RMS(bonds)	0.006
RMS(angles)	0.77
Ramachandran favored (%)	1e+02
Ramachandran allowed (%)	0
Ramachandran outliers (%)	0
Rotamer outliers (%)	1.1
Clashscore	2.02
Average B-factor	23.18
macromolecules	21.87
ligands	73.02
solvent	35.61

Supplementary Table 2. A selected subset of top upregulated or downregulated proteins across ATRA, ATO, ATRA+ATO and Pin1 KO samples identified by quantitative proteomic analysis.

Protein	Log_2(ATO/ctrl)	Log_2(ATRA/ctrl)	Log_2(ATO+ATRA/ctrl)	Log_2(CRISPR/ctrl)	Class	Function in cancer
Pin1	0.027570154	-0.220422248	-1.35057482	-4.145800959	Down-regulated	Oncogenic
SPICE1	-1.904235838	-2.173327566	-2.393085534	-2.752804859	Down-regulated	No known cancer connection
POLR2F	-0.265925262	-0.502542125	-0.537786827	-1.559994785	Down-regulated	No known cancer connection
FN1	-1.105216814	-1.939912808	-0.572555698	-1.420923573	Down-regulated	Oncogenic
DMXL1	-1.199655926	-1.0557431	-1.251789777	-1.339240438	Down-regulated	Putative oncogenic
UBAP2L	-0.546418286	-0.536222106	-0.809615642	-1.145971314	Down-regulated	Oncogenic
ALPP	-0.764954043	-0.279077432	-1.809069572	-1.145832763	Down-regulated	Oncogenic
A2M	-1.196670068	-1.053381568	-0.813555982	-0.961850545	Down-regulated	No known cancer connection
HIST2H2BC	-0.42700503	-0.360125183	-0.438578025	-0.853940905	Down-regulated	Oncogenic
PDCL3	-0.172087887	-0.513593606	-0.576026419	-0.748930563	Down-regulated	Oncogenic
DUS2	-0.764589109	-0.526111836	-0.699884977	-0.745307829	Down-regulated	Oncogenic
CIAO1	-0.350895643	-0.616450973	-0.537349298	-0.736408937	Down-regulated	Oncogenic
S100A4	-0.800763663	-1.328639209	-1.282942683	-0.633515215	Down-regulated	Oncogenic
SCO2	-0.725942318	-0.526130227	-0.916836395	-0.629895411	Down-regulated	No known cancer connection
CKB	-0.569449293	-0.692377977	-1.023720351	-0.503741145	Down-regulated	Oncogenic
FLNB	0.653812655	1.001643566	1.083725149	0.39339465	Up-regulated	Tumor suppressive
SLFN5	0.721157805	1.27017307	1.016655381	0.484748353	Up-regulated	Tumor suppressive
SLC30A1	0.472240118	0.696472924	0.41158345	0.63846252	Up-regulated	Putative tumor suppressive
DDR2	1.446064406	0.587156173	0.977239695	0.653473163	Up-regulated	Putative tumor suppressive
EPB41L1	0.859404968	1.071189394	0.64267321	0.771163002	Up-regulated	Putative tumor suppressive
TFRC	0.50195091	0.44308878	0.471474792	0.807965226	Up-regulated	Oncogenic
CLIC4	0.578844893	0.450962191	0.826453632	0.839465092	Up-regulated	Tumor suppressive
PDCD4	0.257363572	0.235681051	0.422623373	0.860846587	Up-regulated	Tumor suppressive
ARMCX3	0.40042969	0.518410522	0.515409634	0.893445179	Up-regulated	Tumor suppressive
PI4K2A	0.498309429	0.32697915	0.394061922	0.908087251	Up-regulated	Tumor suppressive
ITPR2	0.264168182	0.285592122	0.531496606	1.038451813	Up-regulated	Tumor suppressor
FAM129A	2.056101717	1.443539404	2.166966087	1.060228647	Up-regulated	No known cancer connection
TANC1	0.430595398	0.6917085	0.720155002	1.138081517	Up-regulated	No known cancer connection
SLC38A1	0.553150746	0.424132146	0.692265995	1.218934154	Up-regulated	Oncogenic
CSTB	0.60766484	0.557467583	0.670654934	1.235718354	Up-regulated	Tumor suppressive
